# Personality changes during adolescence predict young adult psychosis proneness and mediate gene–environment interplays of schizophrenia risk

**DOI:** 10.1017/S0033291724002198

**Published:** 2024-10

**Authors:** Linda A. Antonucci, Alessandra Raio, Gianluca Christos Kikidis, Alessandro Bertolino, Antonio Rampino, Tobias Banaschewski, Arun L.W. Bokde, Sylvane Desrivières, Herta Flor, Antoine Grigis, Hugh Garavan, Andreas Heinz, Jean-Luc Martinot, Marie-Laure Paillère Martinot, Eric Artiges, Frauke Nees, Dimitri Papadopoulos Orfanos, Luise Poustka, Sarah Hohmann, Juliane H. Fröhner, Michael N. Smolka, Nilakshi Vaidya, Henrik Walter, Robert Whelan, Gunter Schumann, Catharina A. Hartman, Giulio Pergola, the IMAGEN Consortium

**Affiliations:** 1 Department of Translational Biomedicine and Neuroscience – University of Bari Aldo Moro, Bari, Italy; 2 Psychiatry Unit – Policlinico di Bari, Bari, Italy; 3 Department of Child and Adolescent Psychiatry and Psychotherapy, Central Institute of Mental Health, Medical Faculty Mannheim, Heidelberg University, Mannheim, Germany; 4 Discipline of Psychiatry, School of Medicine and Trinity College Institute of Neuroscience, Trinity College Duin, Dublin, Ireland; 5 Centre for Population Neuroscience and Precision Medicine (PONS), Institute of Psychiatry, Psychology & Neuroscience, SGDP Centre, King's College London, UK; 6 Institute of Cognitive and Clinical Neuroscience, Central Institute of Mental Health, Medical Faculty Mannheim, Heidelberg University, Mannheim, Germany; 7 Department of Psychology, School of Social Sciences, University of Mannheim, Mannheim, Germany; 8 NeuroSpin, CEA, Université Paris-Saclay, Gif-sur-Yvette, France; 9 Departments of Psychiatry and Psychology, University of Vermont, Burlington, Vermont, USA; 10 Department of Psychiatry and Psychotherapy CCM, Charité – Universitätsmedizin Berlin, Corporate Member of Freie Universität Berlin, Humboldt-Universität zu Berlin, and Berlin Institute of Health, Berlin, Germany; 11 Institut National de la Santé et de la Recherche Médicale, INSERM U 1299 “Trajectoires développementales & psychiatrie”, University Paris-Saclay, CNRS, France; 12 Ecole Normale Supérieure Paris-Saclay, Centre Borelli; Gif-sur-Yvette, France; 13 Sorbonne University, Department of Child and Adolescent Psychiatry, Pitié-Salpêtrière Hospital, Paris; France; 14 Psychiatry Department, EPS Barthélémy Durand, Etampes; France; 15 Institute of Medical Psychology and Medical Sociology, University Medical Center Schleswig Holstein, Kiel University, Kiel, Germany; 16 Department of Child and Adolescent Psychiatry and Psychotherapy, University Medical Centre Göttingen, Göttingen, Germany; 17 Department of Child and Adolescent Psychiatry, Psychotherapy and Psychosomatics, University Medical Center Hamburg-Eppendorf, Hamburg, Germany; 18 Department of Psychiatry and Neuroimaging Center, Technische Universität Dresden, Dresden, Germany; 19 Centre for Population Neuroscience and Stratified Medicine (PONS), Department of Psychiatry and Neuroscience, Charité Universitätsmedizin Berlin, Germany; 20 School of Psychology and Global Brain Health Institute, Trinity College Dublin, Ireland; 21 Centre for Population Neuroscience and Precision Medicine (PONS), Institute for Science and Technology of Brain-inspired Intelligence (ISTBI), Fudan University, Shanghai, China; 22 Interdisciplinary Center Psychopathology and Emotion regulation, Department of Psychiatry, University Medical Center Groningen, University of Groningen, Groningen, The Netherlands; 23 Lieber Institute for Brain Development, Johns Hopkins Medical Campus, Baltimore, MD, USA; 24 Department of Psychiatry and Behavioral Science – John Hopkins University, Baltimore, MD, USA

**Keywords:** bullying victimization, gene-environment correlations, latent growth curve models, personality, psychosis proneness

## Abstract

**Background:**

Psychotic symptoms in adolescence are associated with social adversity and genetic risk for schizophrenia. This gene–environment interplay may be mediated by personality, which also develops during adolescence. We hypothesized that (i) personality development predicts later Psychosis Proneness Signs (PPS), and (ii) personality traits mediate the association between genetic risk for schizophrenia, social adversities, and psychosis.

**Methods:**

A total of 784 individuals were selected within the IMAGEN cohort (Discovery Sample-DS: 526; Validation Sample-VS: 258); personality was assessed at baseline (13–15 years), follow-up-1 (FU1, 16–17 years), and FU2 (18–20 years). Latent growth curve models served to compute coefficients of individual change across 14 personality variables. A support vector machine algorithm employed these coefficients to predict PPS at FU3 (21–24 years). We computed mediation analyses, including personality-based predictions and self-reported bullying victimization as serial mediators along the pathway between polygenic risk score (PRS) for schizophrenia and FU3 PPS. We replicated the main findings also on 1132 adolescents recruited within the TRAILS cohort.

**Results:**

Growth scores in neuroticism and openness predicted PPS with 65.6% balanced accuracy in the DS, and 69.5% in the *VS* Mediations revealed a significant positive direct effect of PRS on PPS (confidence interval [CI] 0.01–0.15), and an indirect effect, serially mediated by personality-based predictions and victimization (CI 0.006–0.01), replicated in the TRAILS cohort (CI 0.0004–0.004).

**Conclusions:**

Adolescent personality changes may predate future experiences associated with psychosis susceptibility. PPS personality-based predictions mediate the relationship between PRS and victimization toward adult PPS, suggesting that gene–environment correlations proposed for psychosis are partly mediated by personality.

## Introduction

Current research refers to psychosis proneness signs (PPS) as unusual psychotic-like experiences (PLEs), like perceptual abnormalities or persecutory ideation (Bourgin et al., 2020). PPS occur in both at-psychosis-risk (Bonnett, Varese, Smith, Flores, & Yung, 2019) and non-clinical populations (Loch et al., 2011): up to 26.7% of the general population reports at least one PPS (Bourgin et al., 2020), without seeking psychiatric help (Yung et al., 2009). Interestingly, PPS occur more frequently among young individuals (McGrath et al., 2015) until young adulthood. This age stage also represents the typical onset period for psychosis-spectrum disorders, including schizophrenia (Solmi et al., 2022). Although PPS do not meet the threshold for full-blown psychosis (Mark & Toulopoulou, 2016), their occurrence during adolescence may precede later psychotic onset (Nuevo, Van Os, Arango, Chatterji, & Ayuso-Mateos, 2013). When PPS persist into young adulthood, the individual risk for full-blown psychosis increases (McGrath et al., 2016). Thus, PPS represent an early target to investigate how psychosis susceptibility develops over time (Howes & Murray, 2014). Investigating PPS may refine early identification and prediction of individual risk.

In the context of the high schizophrenia heritability, estimated up to 80% (Lichtenstein et al., 2009), exposure to adverse environmental factors contributes to the risk of developing schizophrenia and other psychoses (van Os, Kenis, & Rutten, 2010). For schizophrenia, the gap between the genetic risk explainable in terms of genetic variants and the heritability estimates in twins is remarkable. Thus, disentangling different types of gene-environment interplay (Zwicker, Denovan-Wright, & Uher, 2018) is important for early identification and primary prevention (Pergola, Penzel, Sportelli, & Bertolino, 2023; Plomin, DeFries, & Loehlin, 1977; Uher & Zwicker, 2017): indeed, gene–environment correlations require the environment to enact genetic risk; the environment may, in turn, be modulated by early intervention programs.

Notably, schizophrenia genetic risk loci have previously been associated with ‘neuroticism’ and ‘openness’ personality traits (Smeland et al., 2017). Personality traits are heritable (Vukasović & Bratko, 2015), and they develop during adolescence (Roberts, Caspi, & Moffitt, 2001). Late adolescence is also the period in which PPS typically emerge. Adolescent PPS have been associated with personality characteristics, especially with schizotypal traits (Barrantes-Vidal, Racioppi, & Kwapil, 2020; Fonseca-Pedrero, Ortuno-Sierra, Inchausti, Rodriguez-Testal, & Debbane, 2019). However, prior research did not examine the identified relationships in the frame of gene–environment interplay models to explain psychopathology development. We reasoned that static (e.g. genetics) and dynamic (e.g. personality) *intrinsic* factors might be modulated by *extrinsic* factors (e.g. social adversities) when contributing to PPS. Pergola et al. (2019) already reported evidence of an evocative gene-environment correlation in schizophrenia. In that study, the polygenic risk score (PRS) for schizophrenia (PGC2 wave), cumulating the effects of many risk variants, was associated with the subsequent frequency of sub-clinical psychotic symptoms. This effect was *mediated by* peer and teacher reports of an established schizophrenia environmental factor, i.e. bullying victimization (Armitage et al., 2022; Woolway et al., 2022). However, it is still unexplained how genetic factors could act on the social environment; one hypothesis considers evocative gene-environment correlations (Lella, Antonucci,, & Pergola, 2023). Peer victimization is not necessary or sufficient *per se* for a diagnosis of schizophrenia; it may represent an environmental risk factor potentially correlated with genetic risk for schizophrenia (Woolway et al., 2022). We hypothesized that adolescent personality evolution may interact with such exposure in predicting psychosis vulnerability, and potentially be associated with genetic susceptibility, as well (Millan et al., 2016). Importantly, genetics, social challenges, and personality evolution are intertwined, and their relationship may be non-linear, e.g. detectable only in the presence of high genetic risk (Pergola et al., 2019). Non-linear interactions between multiple variables are best captured by machine learning approaches when aiming to achieve individual-level predictions (Dwyer, Falkai, & Koutsouleris, 2018). Therefore, we aimed to deliver longitudinal calculators of psychosis risk that quantify the predictive potential of individual personality trajectories by combining multivariate and change-tracking techniques (i.e. Latent Growth Curve Models-LGCM).

**Figure 1.**
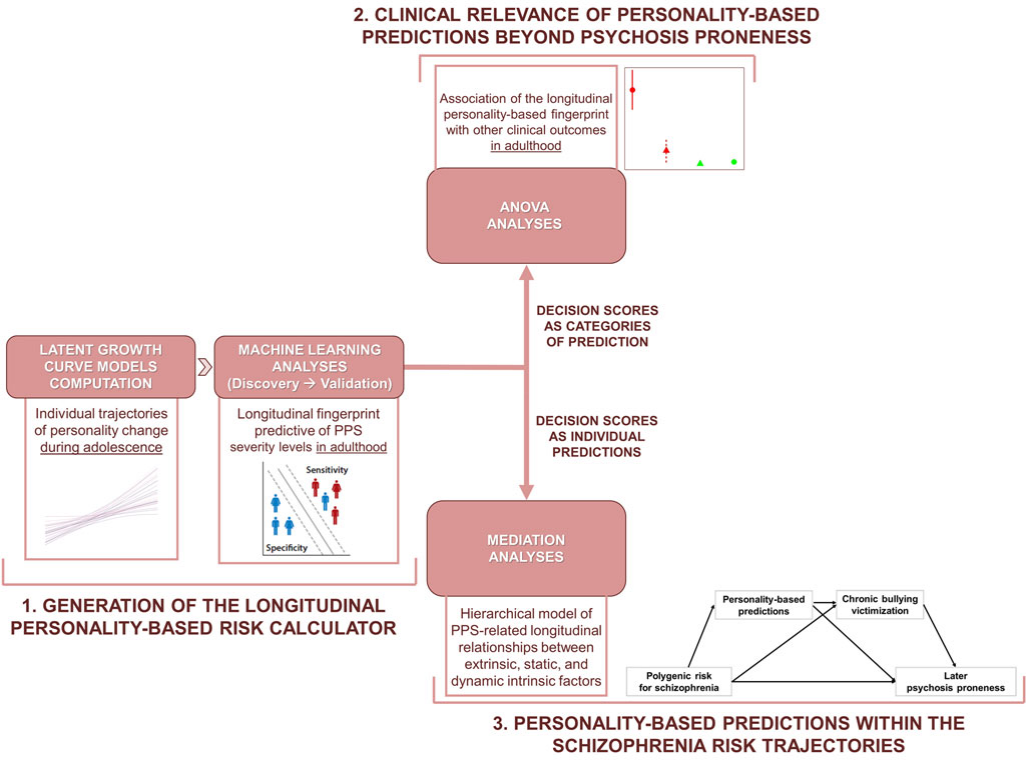
The outline of the study. (1) Individual trajectories of change during adolescence based on personality traits were estimated via latent growth curve models. The two derived trait-related coefficients of change per participant fed a machine learning algorithm as longitudinal predictors of psychosis proneness signs (PPS) in young adulthood. Longitudinal decision scores extracted from the generated models were used: (2) to predict clinical outcomes other than PPS in adulthood; (3) as a longitudinal interface between individual polygenic risk for schizophrenia and bullying victimization across the pathway toward final PPS. (Figure representing machine learning analyses adapted from Dwyer et al., 2018). PPS, Psychosis Proneness Signs.

Here, we aimed to investigate whether personality development during adolescence plays a role in gene–environment interplays predicting subsequent PPS. We analyzed a naturalistic population recruited within the IMAGEN consortium, generating a multivariate risk calculator based on adolescent patterns of personality change aimed at predicting PPS severity in adulthood. Next, we associated our algorithm predictions with the risk of developing emotional and behavioral disorders to explore the wider clinical relevance of our personality-based risk calculator. Finally, we investigated the association of polygenic risk for schizophrenia with subsequent PPS severity. We hypothesized that this relationship was mediated by personality-based multivariate predictions and bullying. To test our findings' generalizability, we replicated the main results on another independent cohort of young individuals (TRAILS). The study is outlined in Fig. 1.

## Methods

### Sample and assessment

We selected 784 individuals recruited within the IMAGEN study (Schumann et al., 2010), from a wider naturalistic cohort of 13-years-old adolescents undergoing four assessment waves, at Baseline (BL) and at two, four, and six years after BL completion (i.e. follow up 1, 2, 3 – FU1, FU2, FU3) (see online Supplementary Information–SI, Section 1). Parents and adolescents gave written consent and verbal assent, respectively. We selected participants based on the full availability of the individual total score for the Community Assessment of Psychic Experiences – 42 (CAPE-42) (Stefanis et al., 2002), used to assess PPS at FU3 as the last available time point (see online SI, Section 2). The IMAGEN cohort was randomly split (2:1 ratio) into a discovery sample (*N* = 526; Table 1A) and a validation sample (N = 258; Table 1B). To test our findings generalizability, we replicated IMAGEN models on an external cohort of 1546 adolescents (Table 1C), selected from the Tracking Adolescents' Individual Lives Survey (TRAILS) study (see online SI, Section 12.1), based on CAPE-42 full data availability at the last accessible time point (wave 3-w3). Details about subsequent steps of participant retention in both cohorts are depicted in a Consort Chart (online Supplementary Figure 1).

**Table 1.** Demographic characteristics of: (**A**) IMAGEN Discovery Sample; (**B**) IMAGEN Validation Sample; (**C**) TRAILS Replication cohort

A. IMAGEN Discovery sample	All subjects (mean ± SD)	High-PPS (mean ± SD)	L-PPS (mean ± SD)	H-PPS *v*. L-PPS T/χ2 (*p*-value)
*N*	526	261	265	n.a.
*Gender ratio* (*M/F*)	235/291	103/158	132/133	5.28 (0.02)*
*Age in years*				
*BL*	13.94 ± 0.39	13.96 ± 1.9	13.91 ± 0.35	1.41 (0.16)
*FU1*	15.99 ± 0.6	15.98 ± 0.62	16.01 ± 0.58	−0.50 (0.61)
*FU2*	18.35 ± 0.6	18.39 ± 0.62	18.32 ± 0.58	1.47 (0.14)
*FU3*	22.04 ± 0.6	22.08 ± 0.67	21.99 ± 0.57	1.76 (0.08)
B. IMAGEN Validation sample	All subjects (mean ± SD)	H-PPS (mean ± SD)	L-PPS (mean ± SD)	H-PPS *v*. L-PPS T/χ2 (*p*-value)
*N*	258	142	116	n.a.
*Gender ratio* (*M/F*)	126/132	63/79	63/53	2.14 (0.14)
*Age*				
*BL*	13.96 ± 0.38	13.94 ± 0.35	13.97 ± 0.38	−0.66 (0.51)
*FU1*	16 ± 0.58	16.01 ± 0.57	16 ± 0.58	0.09 (0.92)
*FU2*	18.37 ± 0.65	18.44 ± 0.68	18.28 ± 0.58	2 (0.046)*
*FU3*	22.02 ± 0.7	22.11 ± 0.64	21.91 ± 0.67	2.45 (0.15)*
C. TRAILS Replication cohort	All subjects (mean ± SD)	H-PPS (mean ± SD)	L-PPS (mean ± SD)	H-PPS *v*. L-PPS T/χ2 (*p*-value)
*N*	1546	750	796	n.a.
*Gender ratio* (*M/F*)	846/700	458/292	388/408	23.17 (<0.001***)
*Age in years*				
*Wave 1*	11.09 ± 0.56	11.08 ± 0.55	11.09 ± 0.56	0.51 (0.61)
*Wave 3*	16.24 ± 0.68	16.21 ± 0.67	16.26 ± 0.70	1.49 (0.13)

BL, Baseline; FU1/FU2/FU3, Follow-Up 1/2/3; H-PPS, Higher-Psychosis Proneness Signs; L-PPS, Lower-Psychosis Proneness Signs; M/F, Male/Female; n.a., not assessed; SD, Standard Deviation. (*) marks *p* < 0.05, (**) marks *p* < 0.01, (***) marks *p* < 0.001.

In both cohorts, each individual was assigned to a higher- or lower-PPS group, based on the median of the FU3 CAPE-42 total score distribution, which for the IMAGEN cohort was calculated in the Discovery sample and used as a cut-off also in the Validation sample (so that data remained independent of cut-off determination). Two-sample *t* tests and *χ*2 tests assessed differences between Higher-PPS and Lower-PPS (IMAGEN Discovery/Validation samples: Table 1A/1B; TRAILS replication cohort: Table 1C; *α* = 0.05).

For each IMAGEN participant, a total of 14 personality scores (listed in online Supplementary Table 1) were computed at BL, FU1, and FU2 based on the items from the following self-report questionnaires (see online SI, Section 3 for details):

- the NEO Five-Factory Inventory (Costa & McCrae, 1992), which assesses personality on five main dimensions (Neuroticism, Extraversion, Openness, Agreeableness, and Conscientiousness), i.e. the so-called Big Five traits (Raad & Perugini, 2002);
- the Temperament and Character Inventory–Revised (Farmer & Goldberg, 2008), based on the Cloninger comprehensive model of temperament and character (Cloninger, Svrakic, & Przybeck, 1993). The IMAGEN version provided measures for Novelty Seeking and its four temperamental subcomponents (exploratory excitability, impulsiveness, extravagance, and disorderliness);
- the substance use risk profile scale (Woicik, Stewart, Pihl, & Conrod, 2009), investigating the role of our main personality traits (Hopelessness, Anxiety Sensitivity, Impulsivity, Sensation Seeking) as potential risk factors for addictive behaviors and co-morbid psychopathology development.

Descriptive statistics and between-group comparisons, performed via two-sample *t* tests (*α* = 0.05), are reported for each selected score at each time point in online Supplementary Table 2.

The three IMAGEN waves of personality data collection were used as input variables to estimate individual trajectories of change in both the discovery and validation samples (see Methods, Section ‘Latent Growth Curve Models pipeline’).

In the TRAILS cohort, a total of 12 personality variables (listed in online Supplementary Table 13), including both w3 cross-sectional and w3-w1 longitudinal scores (see online SI, Section 12.2), were analyzed through the very same machine learning pipeline implemented on IMAGEN data (see Methods, Section ‘Machine learning pipeline’).

### Generation of the longitudinal personality-based risk calculator

#### Latent growth curve models pipeline

To compute personality-based trajectories of individual change over time, we implemented LGCMs (Burant, 2016) via the R statistics (https://cran.r-project.org/) ‘lavaan’ package. A total of 14 LGCMs were computed separately in both Discovery and Validation samples, one for each personality variable assessed at BL, FU1, and FU2 (listed in online Supplementary Table 1). Incomplete personality information at FU1 (missing data proportion: 7.03–9.33%) was handled via full information maximum likelihood (Allison, 1987; Felt, Depaoli, & Tiemensma, 2017). FU3 personality information was excluded from LGCMs computation so that PPS outcomes were solely predicted based on prior time points.

LGCMs computation details are reported in online SI, Section 4.1. Details about goodness-of-fit indices and metrics are reported in online SI, Section 4.2.

From each of the 14 general LGCMs implemented in both the discovery and validation samples, two individual latent coefficients of change (i.e. an intercept factor and a slope factor for each participant) were derived. The 28 personality-based coefficients of change estimated per participant fed a supervised machine learning algorithm built to predict PPS severity levels at FU3 (see Methods, 2.2.2).

#### Machine learning pipeline

We carried out our machine learning analytic strategy via NeuroMiner, version 1.05 (www.pronia.eu/neurominer/) (Antonucci et al., 2020; Koutsouleris et al., 2016). The 28 individual personality-based coefficients of change computed via LGCMs (see Methods, Section ‘Latent Growth Curve Models pipeline’) fed a Support Vector Machine algorithm aimed at predicting Higher- v. Lower-PPS at FU3 in our Discovery sample. We adopted a repeated nested cross-validation design (Antonucci et al., 2021; Koutsouleris et al., 2021) (see online SI, Section 5 for details) to generate a multivariate personality-based longitudinal risk calculator of psychosis proneness. Permutation analyses served to assign statistical significance to both PPS and extra labels prediction performance (Golland & Fischl, 2003; Koutsouleris et al., 2016) (see online SI, Section 6), measured through balanced accuracy (BAC). To assess the generalizability of our personality-based risk calculator, we applied the discovery prediction model to our independent validation sample via out-of-sample cross-validation (OOCV) (Antonucci et al., 2021; Haas et al., 2021).

To verify that generated predictions were not affected by potential confounds, we correlated individual prediction scores extracted from the discovery risk calculator with BL measures for cognitive performance and substance use (see online SI, Section 5.1. for details). All significant *p* values were <0.05, false discovery rate (FDR)-corrected (Benjamini & Hochberg, 1995).

### Personality-based predictions beyond psychosis proneness symptoms

To investigate whether the predictive performance of our personality-based risk calculator was associated with clinical readouts outside PPS, we used ANOVAs on four different categories of prediction (i.e. two groups of correctly predicted v. two groups of misclassified individuals for higher- or lower-PPS) and the risk to develop emotional, conduct, hyperactivity and any other disorders at FU3. Such risk was estimated for individuals included in both the discovery and validation samples through the Development and Well-being Assessment Algorithm (Goodman, Ford, Richards, Gatward, & Meltzer, 2000) from the Strength and Difficulties Questionnaire items (Goodman, 1997). For details, see online SI, Sections 7,8.

### Personality-based predictions within the schizophrenia risk trajectories

To assess the total effect between predictor and outcome, we preliminarily tested the association between polygenic liability for several psychiatric conditions and psychosis proneness via correlation analyses (see online SI, Section 11); then, we investigated how putative relationships between polygenic risk for schizophrenia, exposure to victimization, and personality-based predictions may influence PPS. To this aim, we carried out a serial mediation analysis and two moderated mediation analyses via R statistics, using PROCESS Macro v. 4 (Hayes, 2017) (see online SI, Section 11 for details). All the models, depicted in online Supplementary Figure 2, were implemented only on 653 individuals with available genetic data out of the 784 included participants (see online Supplementary Table 3 for descriptive statistics). Individual genetic risk for schizophrenia was estimated using genetic data to calculate a PRS for schizophrenia (Purcell et al., 2009; Pergola et al., 2019) (see online SI, Section 9). To stratify individuals for chronic victimization, we computed the rank product of victimization total scores at BL, FU1, and FU2 (listed in online Supplementary Table 1, descriptive statistics in online Supplementary Table 4), collected through the Bully Questionnaire (details in online SI, Section 10). This score entered in our mediation framework as a second-order mediator or a moderator factor.

To test the modeled pathway generalizability, the very same serial mediation design was implemented on 1132 TRAILS individuals with available genetic data out of the 1546 included participants (online Supplementary Table 15). We generated two different models, respectively including the rank product of victimization scores at w2 and w3 as reported:

- by children via the Youth Self Report (Achenbach & Rescorla, 2006);
- by parents via the Child Behaviour Checklist (Achenbach & Rescorla, 2006).

Complete details are reported in online SI, Section 12.2 and 12.3.

## Results

### Demographic differences between samples

Demographic characteristics are separately reported for IMAGEN discovery and validation samples and for TRAILS replication cohort. The discovery sample featured a higher proportion of females in H-PPS than in L-PPS (χ^2^ = 5.28; *p* = 0.02) (Table 1A), whereas the TRAILS replication cohort featured a lower proportion (χ^2^ = 23.17; *p* < 0.001) (Table 1C). The validation sample included slightly older individuals in H-PPS than in L-PPS at FU2 (t = 2; *p* = 0.046) and at FU3 (t = 2.45; *p* = 0.015) (Table 1B).

### Machine learning results

**Figure 2.**
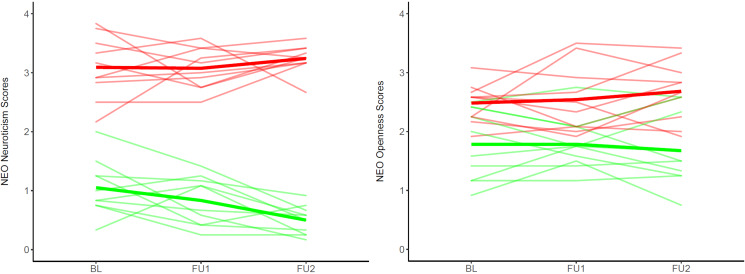
Raw trajectories of change over time for NEO neuroticism scores (left panel) and NEO openness scores (right panel) for the first ten individuals from the upper and the lower limits of the Ensemble Probability Prediction (EPP) scores distribution: notably, as an EPP score from 0.95 above estimated a probability to be assigned to the Higher-Pyschosis Proneness Signs (PPS) class in the 95% of the generated models, we considered such individuals as highly prototypical for such severity class (red lines; the red line in bold depicts the group mean trajectory); as an EPP score from 0.5 below estimated a probability to be assigned to the Higher-PPS class in the 5% of the generated models, we considered such individuals as highly prototypical of the Lower-PPS severity class (green lines; the green line in bold depicts the group mean trajectory) BL, Baseline; FU1, Follow-Up 1; FU2, Follow-Up 2; NEO, NEO Five Factor Inventory.

In the discovery sample, the risk calculator based on the 28 individual personality coefficients of change predicted higher-PPS v. lower-PPS at FU3 with a cross-validated BAC of 65.6% (*p* = 0.01) (detailed metrics in Table 2). Only PPS predictions showed a significant permuted *p*-value (*p* < 0.05). Features with the highest probability of being selected for prediction included individual intercept coefficients for neuroticism, followed by individual slope and intercept coefficients for openness (online Supplementary Figure 3). Figure 2 shows raw trajectories of change over time for neuroticism and openness for the first 10 individuals from the upper ⩾0.95 and the lower ⩽0.05 limits of the ensemble prediction probability scores distribution: the most prototypical individuals from, respectively, the higher-PPS and the lower-PPS prediction class, showed opposite longitudinal patterns (i.e. increasing *v.* decreasing) for neuroticism, whereas they showed only mean differences between higher *v.* lower openness levels over time.

**Table 2.** Validated performance of the personality-based risk calculator predicting Higher- *v*. Lower Psychosis Proneness Signs (PPS) in both Discovery and Validation samples

Higher-PPS *v*. lower-PPS	True negatives	True positives	False negatives	False positives	Sensitivity	Specificity	Balanced accuracy (%)	Area under the curve	Positive Predictive Value	Negative Predictive Value	Positive Likelihood Ratio	Permuted p-value
Discovery sample	159	186	79	102	70.2	60.9	65.6	0.71	64.6	66.8	1.8	0.01
Validation sample	93	85	31	49	73.3	65.5	69.4	0.72	63.4	75	2.1	n.a.

OOCV results (Methods, Section ‘Machine learning pipeline’) revealed that the discovery risk calculator showed high personality-based prediction performance also when applied to validation unseen individuals (BAC: 69.5%; detailed metrics in Table 2). Neither the discovery nor the validation predictions demonstrated inflation due to information leakage (see online SI, Section 5.2. for details). No significant association between prediction scores from the discovery risk calculator and scores at BL for cognitive performance (online Supplementary Table 8) and substance use (online Supplementary Table 9) emerged, suggesting that predictions were not affected by neuropsychological or substance use-related variables.

### Personality-based predictions beyond psychosis proneness symptoms

In both discovery and validation samples, ANOVAs revealed a significant main effect of the prediction rate on scores for emotional and hyperactivity disorders risk at FU3 (all *p* < 0.001, specific *F*-statistics reported in online Supplementary Figure 4). Only individuals correctly predicted by the algorithm as Higher-PPS at FU3 also showed significantly higher risk scores at the same time point: specifically, this pattern occurred only for emotional disorders risk in the Discovery sample (online Supplementary Figure 4A) and for both emotional and hyperactivity disorders risk in the Validation sample (online Supplementary Figure 4C and 4D) (all *p* < 0.001). Only in the Discovery sample, correctly predicted and misclassified Higher-PPS individuals did not differ from each other in terms of estimated risk for hyperactivity disorders, both showing significantly higher scores at FU3 (online Supplementary Figure 4B) (all *p* < 0.05). No significant effect of the prediction index on scores of estimated risk for conduct (Discovery F = 0.68; *p* = 0.56; Validation F = 0.21; *p* = 0.88) or other disorders (Discovery F = 0.84; *p* = 0.72; Validation F = 0.32; *p* = 0.61) emerged at FU3.

### Personality-based predictions within the schizophrenia risk trajectories

**Figure 3.**
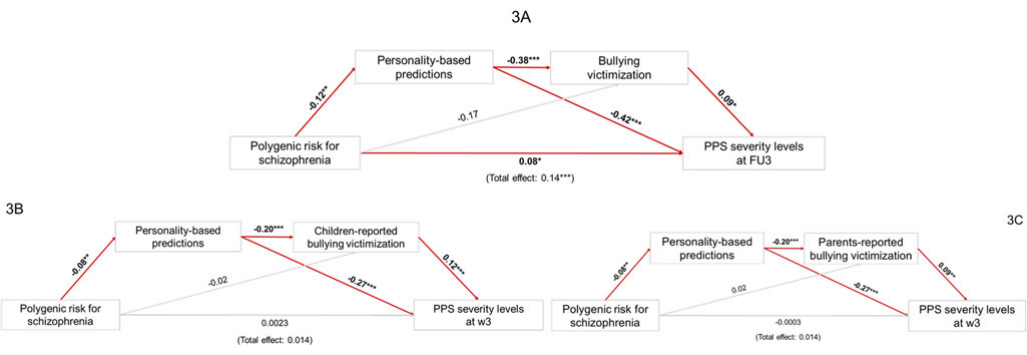
Findings from the serial mediation models, investigating the role of personality-based machine learning predictions and the rank product of Bullying Victimization (BV) within the pathway between polygenic risk for schizophrenia and final Psychosis Proneness Signs. Figure 3A depicted the model generated on IMAGEN data, Fig. 3B and 3C depicted replication models generated on TRAILS data, respectively including children-reported and parents-reported BV information. Direct effects (standardized coefficients) are shown. Red arrows represent relationships returning significant direct effects. The grey arrows represent not significant direct effects. Indirect effects for each model are reported in online Supplementary Table 10 (IMAGEN model), 17, and 18 (TRAILS replication models). FU3, Follow-Up 3; PPS, Psychosis Proneness Signs; w3, wave 3. *marks *p* < 0.05; **marks *p* < 0.01; ***marks *p* < 0.001.

Our preliminary correlation analyses indicated that only the PRS for schizophrenia was significantly associated with PPS (*ρ* = 0.12; *p* = 0.008, FDR-corrected). Thus, we included it as the only predictor in our mediation models. As shown in Fig. 3A, for the IMAGEN serial mediation model, the total effect of the schizophrenia PRS on FU3 PPS severity levels was significant (*β* = 0.14; *p* < 0.001; bootstrapped 95% CI 0.06–0.22). Moreover, the direct effect of the schizophrenia PRS on FU3 PPS severity levels was significant when both the mediators were taken into account (*β* = 0.08; *p* = 0.02; bootstrapped 95% CI 0.01–0.15; 57.1% of the total effect explained (Fairchild, Mackinnon, Taborga, & Taylor, 2009; VanderWeele, 2013)). The remaining 42.9% of the total effect of the schizophrenia PRS on PPS was funneled in an indirect effect. As shown in online Supplementary Table 10, a significant indirect effect emerged within the serial mediation pathway including both personality-based predictions and victimization (*β* = 0.004, bootstrapped 95% CI 0.0006–0.01; 2.8% of effect explained). Also the pathway including only personality-based predictions showed a significant indirect effect, confirming the association discovered via machine learning (*β* = 0.05, bootstrapped 95% CI 0.02–0.09; 35.7% of effect explained). Instead, no significant indirect effect emerged when victimization was considered as the only mediator across the PRS-PPS pathway (*β* = −0.002, bootstrapped 95% CI 0.004−0.01), suggesting the relevance of personality scores in the serial mediation. No moderation effects from victimization emerged on the pathways toward final PPS (see online SI, Section 11 for detailed results).

In the replication on TRAILS data, as shown in online Supplementary Table 17 and 18, we found a significant indirect effect within the serial mediation pathway including both personality-based predictions and victimization, independently of the informants (Fig. 3B model: *β* = 0.002, bootstrapped 95% CI 0.0004–0.004; 20% of variance explained; Figure 3C model: *β* = 0.001, bootstrapped 95% CI 0.0002–0.003; 10% of variance explained). Moreover, the pathway including only personality-based predictions showed a significant indirect effect in both serial mediation models (Fig. 3B model: *β* = 0.022, bootstrapped 95% CI 0.005–0.04; Figure 3C model: *β* = 0.02, bootstrapped 95% CI 0.006–0.04). On the other hand, no significant indirect effect emerged when victimization was considered as the only mediator across the PRS-PPS pathway (Fig. 3B model: *β* = −0.003, bootstrapped 95% CI −0.01 to −0.003; Figure 3C model: *β* = 0.002, bootstrapped 95% CI −0.003 to −0.008).

## Discussion

This study aimed to investigate whether patterns of neurodevelopmental changes in dynamic intrinsic factors like personality may predict adult PPS and whether this prediction ability has a role in gene-environment interplays relevant to schizophrenia. After separating our population based on PPS severity levels, significant between-group differences for raw personality features emerged already at the cross-sectional level. However, findings from our longitudinal risk calculator showed that, in the Discovery sample, higher and lower PPS levels in young adulthood can be accurately predicted through personality and temperament trajectories of change across adolescence. Interestingly, these factors, traditionally considered relatively stable (Hampson & Goldberg, 2006), have been recently reconceptualized as sensitive to change and adaptation, especially throughout adolescence (Caspi, Roberts, & Shiner, 2005; Durbin et al., 2016; Zohar, Zwir, Wang, Cloninger, & Anokhin, 2019). Our longitudinal fingerprint supports this notion and, providing individual predictions, complements group-level cross-sectional evidence of associations between PPS in healthy adolescents and personality (Wiltink et al., 2015) or temperament (Nitzburg et al., 2016). The good validation performance of our risk calculator, when applied to unseen individuals, corroborates its generalizability potential. Indeed, the validation BAC of 69.5% is consistent with the standards reported in previous meta-analyses on machine learning prognostic models built on at-risk individuals (Sanfelici, Dwyer, Antonucci, & Koutsouleris, 2020).

Neuroticism (i.e. the vulnerability to emotional instability and negative emotionality (Begemann, Boyette,, Kwast,, & Sommer, 2020)) and openness (i.e. the availability to new ideas and experiences (Begemann et al., 2020)) emerged as the two personality traits most contributing to longitudinal predictions of PPS levels. Their prominent role in our algorithm is consistent with large-scale studies identifying genetic risk loci shared between schizophrenia and these two personality traits (Lo et al., 2017; Smeland et al., 2017). Accordingly, these traits are associated with psychosis-related manifestations (i.e. symptoms or subclinical PPS severity, and perceived quality of life) in clinical (Franquillo et al., 2021) and healthy populations (Wiltink et al., 2015). Notably, the evolution of these traits over adolescence contributed to the predictions, suggesting that tracking these traits over time enhances early identification. When we compared the most prototypical Higher-PPS and Lower-PPS individuals, the strict separation of longitudinal neuroticism trajectories between the two groups suggested that individuals with higher starting neuroticism scores, and consistently high scores over time, have higher future risk for psychosis. Higher neuroticism is frequently self-reported by individuals prone to negative emotionality and distress (Boyette et al., 2013). This trait may represent a trans-diagnostic risk factor for future psychopathology. High scores on openness have been associated with positive schizotypy and magical thinking (Wiltink et al., 2015) in non-clinical samples (Larøi, DeFruyt,, van Os,, Aleman,, & Van der Linden, 2005; Ross, Lutz, & Bailley, 2002). High openness characterized Higher-PPS individuals in our study but evolved in more heterogeneous within- and between-group trajectories over time. In summary, our findings suggest that the evolution of specific personality traits during adolescence might contribute to future PPS. From a clinical perspective, self-reported neuroticism correlates with maladaptive strategies of emotion regulation (e.g. rumination, self-blaming, suppression (Ludwig, Werner, & Lincoln, 2019)). The same phenomena are reported at different stages of psychosis (Chapman et al., 2020; Vines et al., 2022) and associated with delusion severity in psychotic patients (Garety et al., 2005). It follows that psychoeducational skills training, promoting effective management of negative emotions in such individuals, might contribute to delaying or softening the negative consequences of PPS.

Interestingly, although our algorithm was specific for PPS predictions, neuroticism and openness changes showed potential clinical relevance also for other risk conditions potentially associated with PPS. Indeed, individuals correctly classified as Higher-PPS based on personality changes consistently showed a significantly higher estimated risk of developing emotional and hyperactivity disorders in young adulthood, as also reported previously (Christiansen et al., 2019; Fox, Sheffield, & Woodward, 2021).

Finally, we integrated our longitudinal personality-based predictions in a serial mediation framework including both genetic and environmental features. We found that PPS predictions based on adolescent personality changes were associated with self-reported victimization, and both prediction and victimization serially mediated the relationship between schizophrenia PRS and adult PPS severity. The schizophrenia PRS was also the only one significantly associated with PPS. We cannot exclude that the large sample size of the latest schizophrenia GWAS and the high heritability of this disorder played a role in such a significant effect. Interestingly, the serial mediation effect also emerged when the same mediation design was replicated on the data collected within the external TRAILS cohort, regardless of the different victimization informants (e.g. children or their parents) (Lella et al., 2023) and further methodological differences, confirming the association discovered via machine learning. It is noteworthy that Pergola et al. (2019) described a similar gene-environment correlation without including personality. That study found a victimization-mediated effect of schizophrenia PRS on the frequency of PPS developed at the last accessible time point, including a correlation between PRS and bullying victimization. The PRS-bullying association in TRAILS was not significant when considering personality as a mediator. This evidence suggests that the mechanism of translating genetic risk into environmental risk involves personality traits and their development during adolescence. The lack of a direct PRS-PPS association in TRAILS, whereas it was significant in IMAGEN, is of interest to future research on the assessment of potential critical periods: indeed, demographic differences between the two cohorts (IMAGEN BL-FU3 age: 13.9–22 y.o.; TRAILS w1-w3 age: 11.1–16.2 y.o.; Table 1) overlap with the mixed results on genetic associations obtained in the ALSPAC cohort when data from 13-year olds were included (Schoeler et al., 2019) or not (Riglin et al., 2019). Genetic effects on social patterns characteristic of adolescence may still be too small for detection with the sample sizes employed here in very young individuals. This age specificity would be consistent with other evidence of effects emerging in functional brain imaging only in later adolescence (Passiatore et al., 2023; Zalesky et al., 2015). Nonetheless, the successful replication of the rest of the pathways within the model speaks in favor of its validity and generalizability.

In summary, our results explain the previously reported link (Pergola et al., 2019) between victimization and genetic risk to develop later PPS as a mechanism based on personality. While the PRS of the risk carriers cannot influence the behavior of other individuals, it can influence personality traits and thus the behavioral manifestation of risk, hence explaining the significant associations with victimization. Personality appears as a privileged interface between the polygenic risk for schizophrenia and social adversities. Genetic risk carriers express personality traits predictive of PPS and also suffer a greater environmental burden. This hypothesis was further corroborated by the peer victimization significance as mediator and not moderator, tested on the main IMAGEN cohort data, at variance with previous evidence (Guloksuz et al., 2019). Thus, the environment in this model amplifies genetic risk as in an evocative gene-environment correlation framework, whereby the reactions of the peers to heritable traits expressed by the carrier enhance risk. The small indirect effect on final PPS captured by the risk pathway, including personality and victimization, suggests that the measures of social environment employed picked up only a very modest portion of the environmental contribution to PPS. Thus, while victimization accounts only for a minor proportion of PPS risk, other gene-environment correlation mechanisms may contribute to reduce the heritability gap, identify pathways of genetic risk to environmental exposure, and devise personalized interventions to foster resilience.

## Limitations

To the best of our knowledge, our model of gene–environment correlation informed by personality is the first of its kind. Thus, despite the stringent cross-validation strategy employed and the external replication of our results implemented on TRAILS data, validations in wider naturalistic populations are needed to fulfill generalizability requirements and findings translation into clinical practice (Sanfelici et al., 2020). As bullying is not the only social stressor potentially contributing to psychosis risk, future replications might model PPS vulnerability pathways including additional core factors of social adversity, such as childhood trauma, discrimination, and exclusion (Varchmin, Montag, Treusch, Kaminski, & Heinz, 2021). Additional replications are also recommended for data collections that provide (i) more than three time points to model nonlinear personality change (Durbin et al., 2016), and (ii) a more extensive personality assessment, tapping into traits based on different personality theories.

## Conclusions

Our findings outline a dynamic model of PPS risk development based on a ‘personality-enriched’ gene-environment correlation pathway: genetic factors may act on PPS through an effect on adolescent personality changes, which in turn could modulate the exposure to negative social interactions. For this reason, our findings highlight the importance of monitoring the evolution of personality traits over time. We found that the increase or stability of high neuroticism and openness across adolescence are relevant to future psychosis proneness. Personality development is strictly entangled with environmental challenges. Thus, we envision that real-world programs aimed at softening the downstream effects of an increased genetic risk for psychosis might be oriented toward (i) early identification and management of maladaptive social environments during adolescence and (ii) promoting adaptation to social adversities in subjects with high neuroticism and openness, particularly when victimized by peers.

## Disclosures

Prof Pergola received lecture fees from Lundbeck. Prof. Bertolino received consulting fees from Biogen and lecture fees from Otsuka, Janssen, and Lundbeck. Prof. Rampino received travel fees from Lundbeck. Dr Banaschewski served in an advisory or consultancy role for eye level, Infectopharm, Lundbeck, Medice, Neurim Pharmaceuticals, Oberberg GmbH, Roche, and Takeda. He received conference support or speaker's fee by Janssen, Medice and Takeda. He received royalties from Hogrefe, Kohlhammer, CIP Medien, and Oxford University Press; the present work is unrelated to these relationships. Dr Poustka served in an advisory or consultancy role for Roche and Viforpharm and received speaker's fee by Shire. She received royalties from Hogrefe, Kohlhammer and Schattauer. The present work is unrelated to the above grants and relationships. The other authors report no biomedical financial interests or potential conflicts of interest.

## Supporting information

Antonucci et al. supplementary material Antonucci et al. supplementary material

## Supplementary material

The supplementary material for this article can be found at https://doi.org/10.1017/S0033291724002198.
