# Supplementary material for: Personality changes during adolescence predict young adult psychosis proneness and mediate gene–environment interplays of schizophrenia risk
Source: Psychol Med. 2024 Oct 28;54(14):3838–48. doi: 10.1017/S0033291724002198 (PMC11578906; doi:10.1017/S0033291724002198)
Supplement: Antonucci et al. supplementary material [file S0033291724002198sup001.docx]

**SUPPLEMENTARY INFORMATION**

**1. Participants and sample determination**

We selected a total of 784 individuals from the naturalistic cohort of participants recruited within the IMAGEN study, a multi-site, multi-national longitudinal project, whose detailed recruitment and research procedures have been published elsewhere (Schumann et al., 2010). This collaborative effort between eight European sites includes an overall cohort of 14-year-old adolescents undergoing 4 waves of assessment for different data domains (bio-samples, brain imaging, clinical characteristics, and functioning data), at Baseline (BL) and at 2, 4, and 6 years after BL completion (i.e., Follow Up 1-FU1, Follow-Up 2-FU2 and Follow-Up 3-FU3 respectively). The IMAGEN study protocol was approved by the KCL (King’s College London) College Research Ethics Committee CREC/06/07-71 and by local ethics research committees at each site. Parents and adolescents gave written consent and verbal assent, respectively. To properly assess Psychosis Proneness Signs (PPS) as the main outcome variable of our study, we selected participants based on the availability of the individual total score for the Community Assessment of Psychic Experiences – 42 (CAPE-42) (Stefanis et al., 2002) at the last available time point (TP), i.e., FU3 (see Supplementary Information, SI – Section 2, for further details about CAPE-42).

At first, the cohort of 784 selected individuals was randomly split, with a 2:1 ratio, into a Discovery sample (see Main Text, Table 1A) and a Validation sample (see Main Text, Table 1B). Each individual was defined as having higher or lower PPS, based on the FU3 CAPE-42 total score. Specifically, the median of the total CAPE-42 score was calculated in the Discovery sample, and this value was used as a cut-off also in the Validation sample. Then, individuals in both the Discovery and Validation samples having a total CAPE-42 value higher than the median were assigned to the Higher-PPS group, while those with a score lower than the median were assigned to the Lower-PPS group. Details about subsequent steps of participant retention in the IMAGEN cohort are depicted in a Consort Chart (Supplementary Figure 1). Two-sample t-tests and χ2 tests were employed to investigate potential demographic and clinical differences between Higher-PPS and Lower-PPS within Discovery (see Main Text, Table 1A) and Validation (see Main Text, Table 1B) cohorts. For all tests, significance was set at α=.05.

**2. The Community Assessment of Psychic Experiences – 42**

To properly assess PPS as the main target variable of interest for our study, we determined our overall 784 cohort of participants based on the availability of individual total scores for the CAPE-42 (Stefanis et al., 2002) at FU3.

The CAPE-42 is a 42-item self-report questionnaire measuring positive and negative psychotic symptoms and depressive symptoms on a two-dimensional scale. The first-dimension measures the

frequency of symptoms on a four-point scale of ‘never’ = 1, ‘sometimes’ = 2, ‘often’ = 3, and ‘nearly always’ = 4, and the second dimension measures the degree of distress caused by the experience: ‘not distressed’ = 1, ‘a bit distressed’ = 2, ‘quite distressed’ = 3 and ‘very distressed’ = 4. The total score ranges from 42 to 168 on both dimensions. The positive subscale counts 20 items (range 20 -80 on both dimensions), the negative subscale 14 items (range 14 – 56 on both dimensions) and the depressive subscale 8 items (range 8 – 32 on both dimensions). The CAPE-42 has been designed to assess lifetime psychotic experiences in the general population.

**3. Measures of assessment for personality, temperament and character**

According to both the item availability and the IMAGEN consortium standardized rules for scoring, a total of 14 personality scores, all collected at BL, FU1, and FU2 were computed for each individual. Personality, temperament, and character traits were assessed in both Discovery and Validation individuals through the three following self-report questionnaires:

- the NEO Five-Factory Inventory (NEO-FFI) (Costa & McCrae, 1992) represents the short form of the 240-item extensive NEO-Personality Inventory-Revised. This NEO-FFI version consists of 60 items that can be allocated to 5 subscales of 12 items each, rated from the participants on a 5-point Likert scale, from 1=‘strongly disagree’ to 5=’strongly agree'. This way participants can provide a quicker and accurate self-report measure of personality on five main dimensions, i.e., the so-called Big Five personality traits (Raad & Perugini, 2002), including Neuroticism, Extraversion, Openness, Agreeableness, and Conscientiousness. Specifically, Conscientiousness refers the extent of an individual that is organized, works hard, stays on task, and perseveres to finish the job; Extraversion refers to the extent of an individual to be outgoing, assertive, friendly, and active; Agreeableness reflects the extent of an individual to be cooperative, trusting, polite, and compassionate; Neuroticism describes the extent that someone worries, and is irritable, or easily stressed and, finally, the Openness to Experience refers to the extent of an individual to be curious, imaginative, flexible, and interested in trying new things; from the NEO-FFI we computed and selected **5 overall personality mean scores,** one per each of the so-called Big-Five model personality traits;
- the Temperament and Character Inventory-Revised version (TCI-R) (Farmer & Goldberg, 2008). The TCI-R is a self-administered dimensional questionnaire developed to evaluate 7 basic dimensions of personality as it develops and widely vary within individuals in the general population according to the Cloninger’s comprehensive biopsychosocial model (Cloninger, Svrakic, & Przybeck, 1993). Cloninger’s personality model includes 4 temperament dimensions (i.e., Novelty seeking, Harm Avoidance, Reward Dependence and Persistence) and 3 character dimensions (i.e., Self-directedness, Cooperativeness and Self-transcendence). In the IMAGEN data collection only the 35 self-reported TCI-R items evaluating the Novelty Seeking, as the tendency to respond impulsively to novel stimuli with active avoidance of frustration, were included to assess lower order trait dimensions more specifically related to disinhibitory psychopathology: participants rated each of the 35 items related to NS on a 5-point Likert scale, with 1=‘definitely false’, 2=’mostly false’, 3=’neither true or false’, 4='mostly true', and 5='definitely true'. Thus, we could compute and select for analysis purposes only **5 temperament summary scores**, i.e., the 4 total scores for Exploratory excitability, Impulsiveness, Extravagance and Disorderliness and the overall Novelty Seeking score to which they contribute as temperament sub-dimensions;
- the Substance Use Risk Profile Scale (SURPS) (Woicik, Stewart, Pihl, & Conrod, 2009) is a self-reported questionnaire largely used in epidemiological and longitudinal designs to investigate the role of 4 main personality traits as potential risk factors for addictive behaviors and co-morbid psychopathology development. Specifically, in the IMAGEN 23-items version of the questionnaire, 7 items concur in measuring hopelessness levels (as the tendency to develop bleak expectations about oneself and the future), 5 items concur in measuring anxiety sensitivity levels (as the fear of anxiety-related physical sensations), 5 items concur in measuring impulsivity levels (as lack of premeditation and difficulties with response inhibition), and 6 items concur in measuring sensation seeking levels (as the need for intense and novel experiences). Participants rate each item on a 4-point Likert scale, with 1= ‘strongly disagree', 2= ‘disagree', 3= ‘agree', 4= ‘strongly agree’. Thus, we could compute and select for analysis purposes **4 summary scores**, one for each assessed risk personality trait.

A complete list of all the scores selected from the three aforementioned self-reported questionnaires is reported in Supplementary Table 1. Descriptive statistics (mean and standard deviation values) and between-groups comparisons (Discovery sample VS. Validation sample), performed via two-sample t-tests and χ2 tests, are reported for each aforementioned score at each selected TP in Supplementary Table 2. For all tests, α = 0.05.

**4. Latent Growth Curve Models**

**4.1. Computation pipeline**

Among the recommended approaches to study individual differences in continuous trajectories of change over time, we choose Latent Growth Curve Models (LGCMs) to investigate the personality longitudinal change during development (Burant, 2016; Gana & Broc, 2019): in LGCMs the term *trajectory* refers broadly to the extent, shape and pattern of change on a particular characteristic over time, established by repeated measurements or observations. Within a group of individuals with different lines of development, each one’s pattern of change on these observed measures can be described as their individual *growth* trajectory (Felt, Depaoli, & Tiemensma, 2017). According to our study aims, we computed, separately for the Discovery and Validation samples, 14 basic LGCMs, one per each personality or temperament variable longitudinally collected and selected from the IMAGEN data collection (see Supplementary Table 1 for a complete list of the variables and SI, Section 2 for further details about the self-reported questionnaires of assessment). Despite the estimated trajectory of personality change can assume different functions, including both linear and not linear increase or decline over time (Felt et al., 2017; Gana & Broc, 2019), we could only implement linear basic LGCMs, because only linear change can be traced across the three selected occasions of measurement (i.e., BL, FU1 and FU2) (Felt et al., 2017). Indeed, to ensure an unbiased prediction of PPS severity levels at the last available time point (TP) (i.e., FU3) in the following ML framework, where we used the personality-based coefficients of change estimated in this phase as predictors (see Methods, Section 2.2.2 for details), we intentionally did not include the fourth wave of collected data in the LGCMs computation. Each of the 14 estimated basic LGCMs provided two latent (i.e., unobserved) growth factors that determine the shape of the growth driving the means, variances and covariances of the repeated observed variables (Walls & Wielt, 2007): the first is the intercept, which represents the true mean starting point of the trajectory; the second factor is the slope, which represents the true mean rate of change over the time interval under observation (Felt et al., 2017). By setting different loadings for each included data point of observed variables, we estimated the intercept (i.e., initial level), constraining factor loadings for each wave of data to 1, so to force them to be equal to the first time period, i.e., the BL; differently, we estimated the slope (i.e., rate of change) as a linear function of time, constraining factor loadings to reflect the timing (in years) of each data collection point as follows: 0 for the BL data collection, 2 for 2 years after BL at FU1, and 4 for 4 years after BL for the FU2. Data were modeled using a maximum likelihood estimator with robust standard errors, which estimates all model parameters simultaneously to maximize the likelihood that the effects estimates are representative of the population effects.

Missing data in the personality data at FU1 were handled within the procedure of LGCMs cross-validation itself, through the Full Information Maximum Likelihood (FIML) method, as the indicated gold standard when Structural Equation Models need to be computed also on incomplete information ((Allison, 1987; Arbuckle, 2013)Felt et al., 2017; Gana, 2016). The FIML method fully uses the available data, including partially missing or fully observed, to produce parameter estimates that maximize the likelihood function (Enders & Bandalos, 2001). Given a dataset Y with N observations, where some value may be missing, the likelihood function is the joint probability of f the observed data given the model parameters θ. The likelihood function for the 𝑖-th observation can be written as


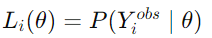


where,
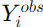
 is the observed part of the data for the i-th case.

Instead of discarding or imputing missing values, FIML uses all available data. For each observation 𝑖, the likelihood is computed based on the observed part of the data
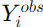
. This involves:

1. computing the covariance matrix and mean vector of the observed data;
2. estimating the likelihood based on the observed data points.

The full likelihood function across all observations is the product of the individual likelihoods:


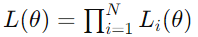


**4.2. Model fit statistics**

Model fit and assessment is typically an important part of implementing any type of LGCM within a Structural Equation Model (SEM)-based framework. Among others, absolute fit statistics are used to determine how well a statistical model reflects the data (Felt et al., 2017), according to specific established cut-off values widely used for SEM, as described by Hu (Hu & Bentler, 1999) and Bentler (Bentler & Bonett, 1980).

Absolute fit measures includes the χ2 goodness-of-fit test, closeness-of-fit measures and badness-of-fit measures (Bentler & Bonett, 1980). Specifically:

- the χ2 goodness-of-fit statistic indicates model adequately reflects the data when the corresponding p-value is above the nominal 0.05 level;
- closeness-of-fit measures include the Comparative Fit Index (CFI) and the Tucker-Lewis Index (TLI); CFI and TLI values close to 1.0 indicate a statistical model that adequately reflects the data. Generally, CFI and TLI values above 0.96 (for CFI) or 0.95 (for TLI) reflect excellent fit, whereas values of 0.90 reflect mediocre fit;
- badness-of-fit measures include the Root Mean Square Error of Approximation (RMSEA) and the Standardized Root Mean Square (SRMS) (i.e., the standardized difference between the observed and predicted correlation); in this case, RMSEA and SRMS values closer to zero indicate a statistical model that adequately reflects the data. Generally, RMSEA and SRMS values of 0.01 reflect excellent fit, values of 0.05 reflect good fit, and values of 0.08 reflect mediocre fit. A not-significant chi-square should also be considered as a further indicator of an estimated model congruent with observed data.

The goodness-of-fit measures reported in Supplementary Table 5 for the implemented LGCMs based on personality-related repeated measures at BL, FU1, and FU2 showed good to excellent fit estimation. Thus, our 14 LGCMs resulted reliable in tracking personality change over time.

**5. Machine learning strategy**

We employed a double cycle, repeated nested cross-validation (CV) framework (Koutsouleris et al., 2016; Koutsouleris et al., 2018), to allow an unbiased estimation of the model’s generalizability, preventing information leaking throughout the strict separation between subjects used for training the models and independent ones used for testing decisions (Ruschhaupt, Huber, Poustka, & Mansmann, 2004). We split the data first into training and test sets on an outer (CV2) cycle, and then we split the resulting training folds again into an inner (CV1), training and test data cycle (Filzmoser, Liebmann, & Varmuza, 2009; Koutsouleris et al., 2016; Varma & Simon, 2006). In our ML framework, parameter optimization is performed within the inner (CV1) cycle, and generalization error estimation is performed only from the outer (CV2) cycle (Ruschhaupt et al., 2004). All model training steps that use group-level statistical procedures (e.g. scaling) occur only in the inner cycle (CV1) training data, whereas the inner cycle test data are used to pick hyperparameter combinations that provide potentially good model generalization capacity. Finally, the outer cycle (CV2) validation data serve exclusively the purpose to measure the models’ generalizability to unseen data. Both in inner (CV1) and outer (CV2) CV levels, we implemented a 10-fold CV cycle. We extended nested CV to repeated nested CV (Vapnik, 1999) at both inner and outer cross-validations cycle by randomly permuting the participants within their groups (number of permutations = 10) and repeating the CV cycle for each of these permutations. Within the CV1, all features underwent a preprocessing pipeline, consisting of the following steps:

1. We scaled each variable to a 0-1 range to remove between features differences effect from the training sample information. The scaling parameters were then applied to the inner and outer CV cycles.

2. To understand the predictive utility of the input variables within the personality-based risk calculator (Antonucci et al., 2020), each feature included underwent a process of forward feature selection (Dwyer, Falkai, & Koutsouleris, 2018; Saeys, Inza, & Larranaga, 2007; Vapnik, 1999), implemented via the NeuroMiner visualization procedure. Specifically, such a procedure selected the most parsimonious subset of features within the variable pool that optimized the algorithm average classification performance across the CV1 training and testing data, providing maximum prognostic performance with the smallest amount of predictors. More specifically, after the data entered a greedy forward search wrapper (Saeys et al., 2007), the feature-related probability of being selected for classification purposes within the inner CV loop was computed for each variable. The wrapper used a linear Support Vector Machine (SVM) (Noble, 2006) to estimate the discriminative value of each variable, extracting the most discriminatory feature and reiterating the process over the remaining variable pool to select the 2nd best-performing variable, which was added to the first one. This reiteration process was repeated until the identification of the optimal variable subspace. To avoid overfitting, we stopped the forward feature when 60% of the variables had been discarded from the feature pool, i.e., when the top 40% of the variables had been extracted by the wrapper.

3. The trained model was then applied to the outer CV cycle (CV2) by preprocessing the best discriminative variables using the learned scaling and determining each validation individual’s outcome class (i.e., Higher-vs. Lower-PPS) through a majority voting procedure across all ensemble models. In other words, in each variable evaluation step in the CV1, the SVM algorithm modeled linear relationships between features and prediction labels (i.e., Higher- vs. Lower-PPS). In the linear kernel space, the separability between Higher-PPS-like and most Lower-PPS-like individuals (i.e., the Support Vectors) was maximized within a hyperplane optimized by the SVM. The trained hyperplane allowed the algorithm to predict subjects' classification of the inner CV1 cycle by projecting its data into the learned kernel space: the estimation of their geometric distance to the decision boundary finally resulted in both an individual decision value and a predicted classification label per participant.

The procedure described above was reiterated for every combination of the SVM parameters related to misclassification cost (C parameters) and kernel width (γ parameters), within a grid defined by the ranges C = [0.125 - 16] and γ = [3.0518-5 - 8]. Within our nested cross-validation framework, an ensemble of 100 models (n=10 repetition x k=10 folds) for each CV2 partition (Antonucci et al., 2020; Antonucci et al., 2021) was created. Performance for the generated models was measured using sensitivity, specificity, Balanced Accuracy (BAC), Area-Under-the Curve (AUC), Positive Predictive Value (PPV), Negative Predictive Value (NPV), and Positive Likelihood Ratio (PLR) based on the probability scores of class membership generated through the ensemble-based majority voting process.

**5.1. Association of prediction performance with neuropsychological or environmental confounds**

To ensure that predictive performance from our longitudinal fingerprint was not affected by any neuropsychological or environmental confound, we conducted Spearman’s correlations between single-subject prediction scores extracted from the personality-based risk calculator decisions and scores at BL for cognitive performance and substance use in the Discovery sample. Indeed, literature on schizophrenia has extensively reported both cognitive deficits and substance abuse as crucial vulnerability factors for psychosis symptoms formation across all the stages of risk (Giuliano et al. 2012; Addington et al. 2014).

To assess cognitive performance, we selected scores from the Passive Avoidance Learning Paradigm (PALP) (Arnett, Newman, & Differences, 2000) and from all the tasks included in the Imagen CANTAB computerized neuropsychological battery (https://www.cambridgecognition.com/cantab/), namely the Pattern Recognition Memory task (PRM), the Affective Go-No-Go task (AGN), the Spatial Working Memory task (SWM), the Cambridge Guessing Task (CGT) and the Rapid Visual Information Processing task (RVP). As measures of alcohol and nicotine consumption, we selected scores from the Alcohol Use Disorders Identification Test (AUDIT) (Saunders, Aasland, Babor, de la Fuente, & Grant, 1993) and the Fagerstrom Test for Nicotine Dependence (FTND) (Heatherton, Kozlowski, Frecker, & Fagerström, 1991) respectively; for drugs other than alcohol, we used all scores about the lifetime substances frequency of use from the “European School Survey Project on Alcohol and Other Drugs” (ESPAD) (Hibell et al., 1997).

Among the different outcome measures provided from each task or self-report questionnaire aimed at evaluating cognitive performance and substance use, we selected only scores with less than 30% of feature-wise missing data (mean and standard deviation values for each selected score are reported in ST6 and ST7).

To fill the missing values in the data, a k-Nearest Neighbor (k-NN) imputation (Troyanskaya et al., 2001; Vapnik, 1999) via R statistics was implemented before running correlation analyses via SPSS (IBM Corp. Released 2016. IBM SPSS Statistics for Windows, Version 24.0. Armonk, NY: IBM Corp.): specifically, for each missing value of a given subject, this procedure allowed to identify a subset of other cases providing available values for both the given variable and all the other variables. This way, a source subset of subjects was sorted according to their similarity with the target one on the basis of the Euclidean distance, and the median of the given variable in the 7 nearest neighbors of given subjects was computed to be used as a reference value to fill the missing one. This process was carried out using the original, non-imputed matrix as a reference and repeated until the imputation for all missing values was completed, filling the respectively computed nearest-neighbor medians.

Results from correlation analyses are fully reported in Supplementary Table 8 and Supplementary Table 9 and revealed no significant association between prediction scores from our personality-based risk calculator and selected variables for cognitive performance and substance use. All significant p values were <0.05, False Discovery Rate (FDR) corrected (Benjamini & Hochberg, 1995).

**5.2. Prediction performance based on cross-validated Latent Growth Curve Models**

Since it was not possible to wrap LGCMs in the NeuroMiner cross-validation setup, we could not *a priori* rule out the possibility that information leakage problems may have inflated the reliability of the generated SVM predictions. To test this hypothesis, we first re-estimated the LGCMs from scratch within the same machine learning cross-validation design described in Section 5; then, we generated a new longitudinal risk calculator based on the cross-validated personality change coefficients.

We summarized in Supplementary Table 19 the performance comparison between the non-cross-validated VS. the cross-validated longitudinal risk calculator.

Findings revealed that Balanced Accuracy across both Discovery and Validation models remains consistent, with negligible differences. Notably, also findings from the procedure of greedy feature selection did not highlight any relevant difference between the two generated algorithms: as clearly depicted in SF7, for the longitudinal risk calculator based on cross-validated coefficients of personality change, the features with a probability of being selected above the chance level (0.5) included again i) individual intercept coefficients for neuroticism, followed by ii) individual slope and iii) intercept coefficients for openness.

Overall, the reported findings demonstrate that generating the SVM model on non-cross-validated coefficients of change did not inflate the PPS predictability potential, presumably because there was no information leakage.

**6. Permutation analysis**

We employed permutation analyses (Golland & Fischl, 2003), to estimate statistical significance for the observed prediction performance of our generated model. Specifically, for each permutation, 1000 random permutations of the outcome labels were performed, and all linear SVM models were retrained in the repeated nested CV design. For each permutation, the predictions of the random models into a permuted prediction ensemble were collected for each outer cycle subject. Thus, a BAC-based null distribution of out-of-training classification performance was built, so that the final calculated significance of the observed out-of-training BAC consisted in the number of events where the permuted out-of-training BAC was higher or equal to the observed BAC divided by the number of performed permutations. The model significance was determined at α=0.05.

We also employed permutation analyses to establish the generalizability of our Discovery personality-based risk calculator into four extra labels other than PPS, each evaluating the vulnerability to different psychosis risk-related conditions at FU3. As already done to dichotomize the CAPE-42 scores distribution (see SI, Section 1 for details), individuals in the Discovery sample were assigned to a Higher VS. Lower risk condition for i) emotional disorders, ii) conduct disorders, iii) hyperactivity disorders, and iv) any other disorder (see SI, Section 8 for details about the assessment tool) on the basis of the median split. None of the four generated extra-models showed significant permuted p-values (all permuted p > 0.05). Thus, findings from such supplementary analyses proved the specificity of our personality-based longitudinal risk calculator for psychosis proneness.

**7. Investigating the relevance of personality-based predictions beyond Psychosis Proneness Signs**

To investigate whether the predictive performance of our longitudinal personality-based risk calculator was associated with clinical readouts outside PPS, we performed ANOVA analyses to test the association between 4 different categories of prediction and the estimated risk to develop emotional, conduct, hyperactivity and any other disorders at FU3 for the individuals included in both the Discovery and Validation samples.

Across the distribution of prediction scores extracted from our personality-based risk calculator, individuals can be grouped in 4 different categories of prediction, i.e., 2 groups of correctly predicted vs. 2 groups of mispredicted individuals for Higher-PPS or Lower-PPS. Specifically:

- the correctly predicted Higher-PPS group included “observed” Higher-PPS individuals that the algorithm correctly predicted as Higher-PPS on the basis of personality coefficients of change;
- the correctly predicted Lower-PPS group included “observed” Lower-PPS individuals that the algorithm correctly predicted as Lower-PPS on the basis of personality coefficients of change;
- the mispredicted Higher-PPS group included “observed” Higher-PPS individuals that the algorithm mispredicted as Lower-PPS on the basis of personality coefficients of change;
- the mispredicted Lower-PPS group included “observed” Lower-PPS individuals that the algorithm mispredicted as Higher-PPS on the basis of personality coefficients of change.

Pairwise post-hoc comparisons between the aforementioned groups were tested through post-hoc analyses, using Benjamini-Hochberg significance correction method.

The 4 scores assessing risk at the last available TP for emotional, conduct, hyperactivity, and any other disorders (listed in Supplementary Table 1) were estimated through the Development and Well-Being Assessment (DAWBA) computer-based package algorithm (Goodman, Ford, Richards, Gatward, & Meltzer, 2000) , which employed for this purpose items from the Strength and Difficulties Questionnaire (SDQ) (Goodman, 1997) (further information about both the SDQ administration through the DAWBA package are reported in SI, Section 8). Descriptive statistics and between-group comparisons, performed via two-sample t-tests and χ2 tests, are reported for each of the 4 aforementioned scores at each selected TP in Supplementary Table 2. For all tests, α=0.05.

**8. Measures of emotional, conduct, hyperactivity, and behavioral disorders**

The risk for emotional, conduct, hyperactivity, and/or any other disorders at the last available TP was assessed in both Discovery and Validation individuals through a computer-administered version of the SDQ (Goodman, 1997). The SDQ consists of 25 items describing positive and negative attributes of children and adolescents that can be allocated to 5 subscales of 5 items each: the emotional symptoms subscale, the conduct problems subscale, the hyperactivity-inattention subscale, the peer problems subscale, and the prosocial behavior subscale. Participants rate each item on a 3-point Likert scale, with 0 = ‘not true’, 1 = ‘somewhat true’, and 2 = ‘certainly true’, the total score per scale ranging 0-10 and a higher score indicating more difficulties. In the IMAGEN assessment protocol, the SDQ items are administered through the DAWBA (Goodman et al., 2000), a computer-based package of rating tools and techniques designed to support clinicians in generating ICD-10 and DSM-IV psychiatric diagnoses on 5-16-year-olds. Thus, as for the DAWBA items, also the answers to questions from SDQ are fed into a computerized algorithm, which predicts how much extreme SDQ scores from the original subscales are predictive of increased probability of clinician-rated emotional, conduct, hyperactivity or any other disorders (Goodman et al., 2000). Specifically, for the SDQ, the DAWBA algorithm provided 4 extra-scores assigning individuals to one of six probability bands, ranging from less than 0.1% likely to more than 70% likely to be rated by the clinician as at risk for emotional, conduct, hyperactivity or any other disorders respectively (where 0.1=<1%; 1=~5%; 2=~3%; 3=~15%; 4=~50%; 5=70%).

**9. Genotype determination and Polygenic Risk Score computation procedure**

Genetic data, extracted from whole-blood samples (~10 mL) and sequenced using Illumina 610Quad v1 chip, were accessed through the IMAGEN consortium (Schumann et al., 2010): Genome-Wide Association Study was performed at the Centre National de Genotype (Evry France, Head M Lathrop) and specific genotyping procedures for IMAGEN are extensively reported elsewhere (Schumann et al., 2010).

For this study purposes, we used genotyped SNPs, which passed standard quality control (n = 477245, list provided by IMAGEN). QC was performed on the cohort using PLINK (version 1.9; <http://pngu.mgh.harvard.edu/purcell/plink/>) (Purcell et al., 2007) according to standards developed by the Psychiatric Genomics Consortium (PGC). We removed:

- SNPs with a missing rate > 5% (before sample removal);
- Subjects with a missing genotype rate > 2%;
- Subjects with autosomal heterozygosity deviation (| Fhet | < 0.2);
- SNPs with a missing rate > 2% (after sample removal);
- SNPs that are not in Hardy-Weinberg equilibrium (HWE: P < 10−6)
- SNPs with a minor allele frequency (MAF) > 1% to exclude rare variants.

Genotype imputation was performed using the pre-phasing/imputation stepwise approach implemented in IMPUTE2 / SHAPEIT (chunk size of 3 Mb and default parameters) and using Phase 3 1000 genome as a reference panel (Delaneau, Marchini, & Zagury, 2011; Howie, Marchini, & Stephens, 2011). After imputation, imputed dosage data for each SNP with imputation quality > 0.9 were used for Polygenic Risk Scores (PRS) calculation. PRS were calculated with PRSice v2 (Choi & O'Reilly, 2019) based on effect sizes of risk loci identified in the most recent schizophrenia PGC3 GWAS (Trubetskoy et al., 2022). Specifically, autosomal SNPs that are not in linkage disequilibrium (R2<0.1) were used to assess the sum of risk alleles weighted by their effect size (natural log of the odds ratio) of association with schizophrenia, at each independent locus across the whole genome, as described elsewhere (International Schizophrenia et al., 2009).

Consistent with the original approach from the Schizophrenia Working Group of the Psychiatric Genomics (Schizophrenia Working Group of the Psychiatric Genomics, 2014) GWAS study, a PRS was calculated using subsets of SNPs (N=23,975) that were significantly associated with schizophrenia (nominal *p* < .05). To control for population stratification in the association analysis, the first 10 principal components of the whole genome data were calculated using SNPRelate v1.3 (Zheng et al., 2012) to be included as nuisance covariates or regressed out for each individual PRS in the implemented SEM models.

**10. Measures for bullying victimization**

Bullying and victimization were assessed over time in the selection of 653 individuals (Supplementary Table 3) with available genetic data through the Bully Questionnaire, a self-report survey developed within the IMAGEN consortium. Questions have been adapted from a questionnaire used in a large international study entitled Health Behaviour in School-aged Children (HBSC) and were initially utilized in the revised Olweus Bully/Victim Questionnaire (Olweus, 1996) and the Youth Risky Behaviour Survey (Brener, Collins, Kann, Warren, & Williams, 1995). The Bully Questionnaire consists of 12 items aimed at assessing how often the participant experienced 12 situations related both to acted bullying behaviors and to suffered victimization in the past 6 months: 6 items refer to 6 different bullying behaviors and can be allocated to the Bullying subscale; the other 6 items refer to 6 different victimization experiences and can be allocated to the Victimization subscale. Participants rate the frequency of each item on a 5-point Likert scale, with 1 = 'None', 2 = 'Only once or twice', 3 = '2 or 3 times a month', 4 = 'About once a week', 5 = 'Several times a week', with a total score per-scale ranging 1-30. According to the consortium standardized rules for the Bully Questionnaire scoring, we could compute and select for analysis purposes **the total summary score for Victimization subscale at BL, FU1, and FU2** (see Supplementary Table 4 for descriptive statistics). Reduced forms of Bullying and Victimization subscales can be obtained, removing from each subscales the two items asking how frequently the participants bullied / have been bullied by a teacher or by a family member.

In order to properly account for repeated measures of bullying victimization, we used R statistics to compute the Rank Product (Breitling, Armengaud, Amtmann, & Herzyk, 2004; Eisinga, Breitling, & Heskes, 2013) of Victimization scores (Victimization RP) as measured by Bully Questionnaire at BL, FU1, and FU2. Specifically, for \(N\) units having \(M\) metrics, \(i = 1 \dots N\), the rank product is defined as:


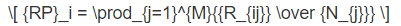


where the rank \(R_{ij} of value \(X_{ij}\) (for unit \(i\) and metric \(j\)) is defined such that ranks vary from 1 (highest) to N (lowest), assuming no ties and no missing data. The terms of the cumulative product are the rank fractions, which under the same assumptions vary from \(\frac{1}{N}\) (highest) to \(1\) (lowest).

**11. Serial and moderated mediation analyses**

Mediation models usually require a significant total effect between predictor and outcome (O'Rourke & MacKinnon, 2015). With the aim to test this requirement, we performed preliminary correlation analyses investigating the association between PPS at FU3 and PRSs for several psychiatric conditions other than schizophrenia. Based on the availability of information from published GWAS, we computed PRSs for ADHD, other psychotic symptoms, autism spectrum disorders, panic disorder, bipolar disorder, major depressive disorder, obsessive-compulsive disorder, and schizophrenia. To properly control for ancestry and different recruitment sites, all the PRSs were marginalized for both the ten genomic eigenvariates and the batch effect before running the analysis. Only the PRS for schizophrenia resulted significantly associated with PPS. Other significant p values were >0.05, FDR-corrected. Thus, only the schizophrenia PRS was included as a predictor in the generated mediation models.

As shown in Supplementary Figure 2, both in the serial mediation (Supplementary Figure 2A) and in the moderated mediation models (Supplementary Figure 2B, Supplementary Figure 2C), personality-based predictions were included as a mediator of the association between the polygenic risk for schizophrenia (measured by PRS) as a predictor and PPS severity levels at FU3 as an outcome variable. In serial mediation, bullying victimization entered the model as a second-order mediator between personality-based prediction and final PPS severity levels. In the two moderated mediation models, bullying victimization was included as a moderator of the pathways from both personality-based predictions (Supplementary Figure 3B) and PRS (Supplementary Figure 3C) toward final PPS severity levels. All the models also included sex and ten genomic eigenvariates to control for ancestry as nuisance covariates. Moreover, before running both the analyses, the schizophrenia PRS was marginalized for batch effect in order to minimize variation between different recruitment sites, and all the variables included (except the nuisance covariates) were mean-centered. A bootstrapping method (with a Bollen-Stine correction to control for deviation from normal distribution) was applied to determine the significance of mediators (A. F. Hayes & Rockwood, 2017; Andrew F Hayes, 2017): specifically, an effect was considered as significant if its 95% bootstrap confidence interval from 5000 bootstrap samples did not include zero (Yu et al., 2022). Direct, indirect, and total effects (i.e., path estimates) of the serial mediation model were examined and reported in Main Text Results, Section 3.4.

For what concerned moderation models results, the direct effect of PRS for schizophrenia persisted when BV was used as a moderator of the pathways from personality-based predictions toward final PPS (Supplementary Figure 5A model: β=0.08, p=0.02, bootstrapped 95% CI=0.01-0.15; Supplementary Figure 5B model: β=0.08, p=0.02; bootstrapped 95% CI=0.01-0.15). No moderation effect from BV emerged on the pathways toward final PPS, neither from personality-based predictions ((β=-0.0002, p =0.99; bootstrapped 95% CI=-0.06-0.06) (Supplementary Table 11, Supplementary Figure 5A) nor from PRS for schizophrenia (β=0.04, p =0.23; bootstrapped 95% CI=-0.02-0.10) (Supplementary Table 12, Supplementary Figure 5B).

**12. Personality-based predictions within the schizophrenia risk trajectories: replication in the TRAILS cohort**

To investigate the generalizability of the putative relationships identified on the IMAGEN cohort data between polygenic risk for schizophrenia, exposure to BV, personality-based predictions and PPS (see Main Text, Section 2.4 and 3.4; SI, Section 11), we replicated the implemented serial mediation design on data from an independent cohort of adolescents selected within the Tracking Adolescents' Individual Lives Survey (TRAILS) study. Detailed information about procedures regarding i) sample determination, ii) measures of assessment selection and iii) data analyses are respectively provided in the following supplementary sections.

**12.1 Participants and sample determination**

We selected an external cohort of 1,546 individuals from the broader population recruited within the TRAILS study. This ongoing multi-disciplinary prospective cohort study, whose detailed recruitment and research procedures have been described elsewhere (Hartman et al., 2022; Huisman et al., 2008), started in 2000 with the aim to investigate mental health and social development from early adolescence into adulthood. The TRAILS consortium includes members of various departments across several universities throughout The Netherlands and recruited an overall cohort of 2,230 adolescents, aged 10-12 y.o. at the baseline wave of assessment, undergoing subsequent measurement waves for different data domains (bio-samples, clinical characteristics, and functioning data) bi- or triennially. Specifically, we obtained access to data from waves 1, 2, and 3 (w1, w2, w3). TRAILS procedures were approved by the Dutch Central Committee on Research Involving Human Subjects and were conducted in accordance with the Declaration of Helsinki, acquiring informed written consent from both adolescents and parents. As already done for the IMAGEN cohort, we selected participants based on the full availability of the individual total score for the CAPE-42 at the last available data wave, i.e. the w3 (see SI – Section 2, for further details about CAPE-42). Each of the 1,546 individuals included in the TRAILS cohort was defined as having higher or lower PPS, based on the median of the w3 CAPE-42 total score distribution, as previously done within the IMAGEN cohort (see SI, Section 1 for further details). Details about subsequent steps of participant retention in the TRAILS cohort are depicted in Supplementary Figure 1. From the two-sample t-tests and χ2 tests, employed to investigate potential demographic differences between Higher-PPS and Lower-PPS within the TRAILS cohort (α=0.05), only a significantly higher proportion of males in Higher-PPS than in Lower-PPS emerged (χ^2^=23.17; p<0.001) (see Main text, Table 1C for further details).

**12.2 Measures of assessment**

Personality, temperament, and character. According to the personality data availability within the TRAILS repository, a total of 12 personality scores were selected for each of the 1,546 individuals. Personality, temperament, and character traits were assessed through the two following questionnaires:

- the Revised NEO Personality Inventory (NEO-PI-R) (Costa Jr & McCrae, 2008) represents the extensive form of the 60-item NEO-FFI used within the IMAGEN consortium to provide personality assessment according to the theoretical framework of the Big Five model (see SI, Section 3). This NEO-PI-R version consists of 240 items, answered by participants on a 5-point Likert scale, from 1=‘strongly disagree’ to 5=’strongly agree', that allows to measure multiple facets related to Neuroticism, Extraversion, Openness to experience, Agreeableness and Conscientiousness. Specifically, in the TRAILS repository, **children-reported NEO-PI-R information** was available **at the w3 only** and included **6 standardized facet scores** we selected, assessing Vulnerability to stress, Excitement seeking, Impulsivity, Assertiveness, Self-discipline, and Hostility;
- the Early Adolescent Temperament Questionnaire – Revised (EATQ-R) (Capaldi & Rothbart, 1992) is a revision of a 1992 instrument developed by Capaldi & Rothbart, designed to specifically assess temperament and self-regulation tapping experiences common to adolescents aged 9-15 y.o. through reports from multiple informants, including both children themselves and their parents. The EATQ-R consists of 62 items, answered by the informant on a 5-point Likert scale, from 1=‘not at all true’ to 5=‘very true’, that contribute to the computation of different summary scores referring each to one of 10 subscales. Specifically, in the TRAILS repository, **parents-report EATQ-R information** was available **at w1 and w3 only** and included, at each wave of assessment, **6 summary scores** evaluating Affiliation (i.e., the desire for warmth and closeness with others, independent of shyness or extraversion), Effortful Control (i.e., the capacity to effectively plan behaviors, and to suppress inappropriate responses), Fear (i.e., the unpleasant affect related to anticipation of distress), Frustration (i.e., the negative affect related to interruption of ongoing tasks or goal blocking), Surgency (i.e., the pleasure derived from activities involving high intensity or novelty) and Shyness (i.e., the behavioral inhibition to novelty and challenge, especially social). In order to enhance the availability of repeated measures for the temperament information, we estimated the between-waves rate of change by computing, for each of the 6 available summary scores, the w3-w1 measurement difference (Δ), selecting the resulting **6 Δ scores** for replication purposes.

A complete list of all the scores selected from the three aforementioned self-reported questionnaires is reported in Supplementary Table 13. Descriptive statistics (mean and standard deviation values) and between-groups comparisons (Higher-VS. Lower-PPS), performed via two-sample t-tests, are reported for each aforementioned score at each selected data wave in Supplementary Table 14. For all tests, α = 0.05.

The total number of 12 personality scores selected per participant (finally including 6 raw scores evaluating cross-sectional personality and 6 estimated Δ scores evaluating temperament change) fed a supervised ML algorithm built to predict high VS. low PPS severity levels at w3, following the very-same pipeline implemented on the main IMAGEN cohort data (see Main Text, Section 2.2.2 and SI, Sections 5 and 6).

Poligenic Risk Score. Individual genetic risk for schizophrenia was estimated using TRAILS genetic data to calculate a PRS for schizophrenia (International Schizophrenia et al., 2009; Pergola et al., 2019), following the very same pipeline implemented on the main IMAGEN cohort data (see SI, Section 9 for a detailed description of the procedure). Specifically, the PRS computation was performed only for 1,132 individuals out of the 1,546 included TRAILS participants, on the basis of genetic information availability (see Supplementary Table 15 for details about demographic and clinical characterization).

Bullying victimization. BV was assessed over time in the selection of 1,132 TRAILS participants with available genetic data, referring to item-specific data reported at w2 and w3 from both children and their parents via Youth Self Report (YSR) and Child Behavior Checklist (CBCL) respectively (see ST16). Both the 112-items YSR and the 113-items CBCL are components of the Achenbach System of Empirically Based Assessment (ASEBA) (Achenbach & Verhulst, 2010), used to detect behavioral and emotional problems in children and adolescents. Both the instruments evaluate and collect information about 8 basic narrow-band syndromes (Anxious-Depressed, Withdrawn/Depressed, Somatic Complaints, Social Problems, Thought Problems, Attention Problems, Rule-Breaking Behavior, and Aggressive Behavior), as grouped in broad-band syndromes (externalizing and internalizing), contributing to a total score evaluating overall behavioral problems. Both YSR children-reported responses and CBCL parents-reported answers are recorded on a 3-point Likert scale (0=‘not true’, 1=’somewhat or sometimes true’, or 2=‘very true or often true’). In the TRAILS version of both instruments, item 38 asks how much children / their parents perceive to be / their children are teased/bullied. In order to properly account for repeated measures of BV, we computed a RP score of children-reported BV (based on answers provided at w2 and w3 to the YSR item-38) and a RP score of parents-reported BV (based on answers provided at w2 and w3 to the CBCL item-38), following the very-same procedure already described in SI, Section 10.

**12.3 Serial mediation analyses**

To replicate on TRAILS cohort data the serial mediation design implemented on the main IMAGEN cohort, we followed the very same analysis pipeline already described in Main Text, Section 2.4 and in SI, Section 11.

Specifically, on the TRAILS data we implemented two different models of serial mediation, according to the different informants reporting information about BV. Consequently, in both the serial mediation models personality-based predictions were included as a first-order mediator of the association between the polygenic risk for schizophrenia (measured by PRS) as a predictor and PPS severity levels at w3 as an outcome variable; additionally, in both the models, BV was included as a second order mediator between personality-based prediction and final PPS severity levels, figuring out within one model as the RP of children-reported measures, within the other one as the RP of parents-reported measures. As Main Text, Section 3.4 reports in detail, we found significant indirect effects within the pathways of serial mediation, involving both personality-based predictions and BV, as well as within the pathways that solely focused on personality-based predictions, regardless of the informants involved. Additionally, the total effect of the schizophrenia PRS on w3 PPS severity levels was not significant for both the implemented models, irrespectively the BV informants were children (Main Text, Figure 3B model: β=0.01; p=0.38; bootstrapped 95% CI=-0.02-0.04) or parents (Main Text, Figure 3C model: β=0.01; p=0.38; bootstrapped 95% CI=-0.02-0.04). Also the direct effect of the schizophrenia PRS on w3 PPS severity levels was not significant for both models (Main Text, Figure 3B model: β=0.002; p=0.88; bootstrapped 95% CI=-0.03-0.03; Main Text, Figure 3C model: β=-0.0003; p=0.98; bootstrapped 95% CI=-0.03-0.03).

**SUPPLEMENTARY REFERENCES**

Achenbach, Thomas M, & Verhulst, F %J Burlington, Vermont. (2010). Achenbach system of empirically based assessment (ASEBA).

Allison, Paul D. (1987). Estimation of linear models with incomplete data. *Sociological methodology*, 71-103.

Antonucci, L. A., Penzel, N., Pergola, G., Kambeitz-Ilankovic, L., Dwyer, D., Kambeitz, J., . . . Koutsouleris, N. (2020). Multivariate classification of schizophrenia and its familial risk based on load-dependent attentional control brain functional connectivity. *Neuropsychopharmacology, 45*(4), 613-621. doi:10.1038/s41386-019-0532-3

Antonucci, L. A., Raio, A., Pergola, G., Gelao, B., Papalino, M., Rampino, A., . . . Bertolino, A. (2021). Machine learning-based ability to classify psychosis and early stages of disease through parenting and attachment-related variables is associated with social cognition. *BMC Psychology, 9*(1), 47. doi:10.1186/s40359-021-00552-3

Arbuckle, James L. (2013). Full information estimation in the presence of incomplete data. In *Advanced structural equation modeling* (pp. 243-277): Psychology Press.

Arnett, Peter A, Newman, Joseph P %J Personality, & Differences, Individual. (2000). Gray's three-arousal model: An empirical investigation. *Personality and Individual Differences, 28*(6), 1171-1189.

Benjamini, Yoav, & Hochberg, Yosef %J Journal of the Royal statistical society: series B. (1995). Controlling the false discovery rate: a practical and powerful approach to multiple testing. *Journal of the Royal Statistical Society: Series B (Methodological), 57*(1), 289-300.

Bentler, Peter M, & Bonett, Douglas G %J Psychological bulletin. (1980). Significance tests and goodness of fit in the analysis of covariance structures. *Psychological Bulletin, 88*(3), 588.

Brener, Nancy D, Collins, Janet L, Kann, Laura, Warren, Charles W, & Williams, Barbara I %J American journal of epidemiology. (1995). Reliability of the youth risk behavior survey questionnaire. *American journal of epidemiology, 141*(6), 575-580.

Burant, C. J. (2016). Latent Growth Curve Models: Tracking Changes Over Time. *International journal of aging & human development, 82*(4), 336-350. doi:10.1177/0091415016641692

Capaldi, Deborah M., & Rothbart, Mary K. (1992). Development and validation of an early adolescent temperament measure. *The Journal of Early Adolescence, 12*(2), 153-173. doi:10.1177/0272431692012002002

Choi, S. W., & O'Reilly, P. F. (2019). PRSice-2: Polygenic Risk Score software for biobank-scale data. *Gigascience, 8*(7). doi:10.1093/gigascience/giz082

Cloninger, C. R., Svrakic, D. M., & Przybeck, T. R. (1993). A psychobiological model of temperament and character. *Archives of general psychiatry, 50*(12), 975-990. doi:10.1001/archpsyc.1993.01820240059008

Costa Jr, Paul T., & McCrae, Robert R. (2008). The Revised NEO Personality Inventory (NEO-PI-R). In *The SAGE handbook of personality theory and assessment, Vol 2: Personality measurement and testing.* (pp. 179-198). Thousand Oaks, CA, US: Sage Publications, Inc.

Costa, Paul T, & McCrae, Robert R %J Psychological assessment. (1992). Normal personality assessment in clinical practice: The NEO Personality Inventory. *Psychological Assessment, 4*(1), 5.

Delaneau, O., Marchini, J., & Zagury, J. F. (2011). A linear complexity phasing method for thousands of genomes. *Nature Methods, 9*(2), 179-181. doi:10.1038/nmeth.1785

Dwyer, D. B., Falkai, P., & Koutsouleris, N. (2018). Machine Learning Approaches for Clinical Psychology and Psychiatry. *Annual review of clinical psychology, 14*, 91-118. doi:10.1146/annurev-clinpsy-032816-045037

Farmer, Richard F, & Goldberg, Lewis R %J Psychological assessment. (2008). A psychometric evaluation of the revised Temperament and Character Inventory (TCI-R) and the TCI-140. *Psychological Assessment, 20*(3), 281.

Felt, J. M., Depaoli, S., & Tiemensma, J. (2017). Latent Growth Curve Models for Biomarkers of the Stress Response. *Frontiers in neuroscience, 11*, 315. doi:10.3389/fnins.2017.00315

Filzmoser, Peter, Liebmann, Bettina, & Varmuza, Kurt %J Journal of Chemometrics: A Journal of the Chemometrics Society. (2009). Repeated double cross validation. *Journal of chemometrics, 23*(4), 160-171.

Gana, Kamel, & Broc, Guillaume. (2019). *Structural equation modeling with lavaan*: John Wiley & Sons.

Golland, P., & Fischl, B. (2003). Permutation tests for classification: towards statistical significance in image-based studies. *Information Processing in Medical Imaging, 18*, 330-341. doi:10.1007/978-3-540-45087-0_28

Goodman, R. (1997). The Strengths and Difficulties Questionnaire: a research note. *Journal of child psychology and psychiatry, and allied disciplines, 38*(5), 581-586. doi:10.1111/j.1469-7610.1997.tb01545.x

Goodman, R., Ford, T., Richards, H., Gatward, R., & Meltzer, H. (2000). The Development and Well-Being Assessment: description and initial validation of an integrated assessment of child and adolescent psychopathology. *Journal of child psychology and psychiatry, and allied disciplines, 41*(5), 645-655.

Hartman, C. A., Richards, J. S., Vrijen, C., Oldehinkel, A. J., Oerlemans, A. M., & Kretschmer, T. (2022). Cohort Profile Update: The TRacking Adolescents' Individual Lives Survey-The Next Generation (TRAILS NEXT). *International journal of epidemiology, 51*(5), e267-e275. doi:10.1093/ije/dyac066

Hayes, A. F., & Rockwood, N. J. (2017). Regression-based statistical mediation and moderation analysis in clinical research: Observations, recommendations, and implementation. *Behaviour research and therapy, 98*, 39-57. doi:10.1016/j.brat.2016.11.001

Hayes, Andrew F. (2017). *Introduction to mediation, moderation, and conditional process analysis: A regression-based approach*: Guilford publications.

Heatherton, T. F., Kozlowski, L. T., Frecker, R. C., & Fagerström, K. O. (1991). The Fagerström Test for Nicotine Dependence: a revision of the Fagerström Tolerance Questionnaire. *British journal of addiction, 86*(9), 1119-1127. doi:10.1111/j.1360-0443.1991.tb01879.x

Hibell, Björn, Andersson, Barbro, Bjarnason, Thoroddur, Kokkevi, Anna, Morgan, Mark, Narusk, Anu, & Ahlström, S. (1997). The 1995 ESPAD report. Alcohol and other drug use among students in 26 European countiries.

Howie, B., Marchini, J., & Stephens, M. (2011). Genotype imputation with thousands of genomes. *G3 (Bethesda), 1*(6), 457-470. doi:10.1534/g3.111.001198

Hu, Li‐tze, & Bentler, Peter M %J Structural equation modeling: a multidisciplinary journal. (1999). Cutoff criteria for fit indexes in covariance structure analysis: Conventional criteria versus new alternatives. *Structural Equation Modeling, 6*(1), 1-55.

Huisman, M., Oldehinkel, A. J., de Winter, A., Minderaa, R. B., de Bildt, A., Huizink, A. C., . . . Ormel, J. (2008). Cohort profile: the Dutch 'TRacking Adolescents' Individual Lives' Survey'; TRAILS. *International journal of epidemiology, 37*(6), 1227-1235. doi:10.1093/ije/dym273

International Schizophrenia, Consortium, Purcell, S. M., Wray, N. R., Stone, J. L., Visscher, P. M., O'Donovan, M. C., . . . Sklar, P. (2009). Common polygenic variation contributes to risk of schizophrenia and bipolar disorder. *Nature, 460*(7256), 748-752. doi:10.1038/nature08185

Koutsouleris, N., Kahn, R. S., Chekroud, A. M., Leucht, S., Falkai, P., Wobrock, T., . . . Hasan, A. (2016). Multisite prediction of 4-week and 52-week treatment outcomes in patients with first-episode psychosis: a machine learning approach. *Lancet Psychiatry, 3*(10), 935-946. doi:10.1016/S2215-0366(16)30171-7

Koutsouleris, N., Kambeitz-Ilankovic, L., Ruhrmann, S., Rosen, M., Ruef, A., Dwyer, D. B., . . . Consortium, Pronia. (2018). Prediction Models of Functional Outcomes for Individuals in the Clinical High-Risk State for Psychosis or With Recent-Onset Depression: A Multimodal, Multisite Machine Learning Analysis. *JAMA Psychiatry, 75*(11), 1156-1172. doi:10.1001/jamapsychiatry.2018.2165

Noble, W. S. (2006). What is a support vector machine? *Nature Biotechnology, 24*(12), 1565-1567. doi:10.1038/nbt1206-1565

O'Rourke, H. P., & MacKinnon, D. P. (2015). When the test of mediation is more powerful than the test of the total effect. *Behavior research methods, 47*(2), 424-442. doi:10.3758/s13428-014-0481-z

Olweus, Dan. (1996). *The revised Olweus bully/victim questionnaire*: University of Bergen, Research Center for Health Promotion.

Purcell, S., Neale, B., Todd-Brown, K., Thomas, L., Ferreira, M. A., Bender, D., . . . Sham, P. C. (2007). PLINK: a tool set for whole-genome association and population-based linkage analyses. *American journal of human genetics, 81*(3), 559-575. doi:10.1086/519795

Raad, Boele de Ed, & Perugini, Marco Ed. (2002). *Big five factor assessment: Introduction*: Hogrefe & Huber Publishers.

Ruschhaupt, M., Huber, W., Poustka, A., & Mansmann, U. (2004). A compendium to ensure computational reproducibility in high-dimensional classification tasks. *Statistical applications in genetics and molecular biology, 3*, Article37. doi:10.2202/1544-6115.1078

Saeys, Y., Inza, I., & Larranaga, P. (2007). A review of feature selection techniques in bioinformatics. *Bioinformatics (Oxford, England), 23*(19), 2507-2517. doi:10.1093/bioinformatics/btm344

Saunders, J. B., Aasland, O. G., Babor, T. F., de la Fuente, J. R., & Grant, M. (1993). Development of the Alcohol Use Disorders Identification Test (AUDIT): WHO Collaborative Project on Early Detection of Persons with Harmful Alcohol Consumption--II. *Addiction, 88*(6), 791-804. doi:10.1111/j.1360-0443.1993.tb02093.x

Schizophrenia Working Group of the Psychiatric Genomics, Consortium. (2014). Biological insights from 108 schizophrenia-associated genetic loci. *Nature, 511*(7510), 421-427. doi:10.1038/nature13595

Schumann, G., Loth, E., Banaschewski, T., Barbot, A., Barker, G., Buchel, C., . . . consortium, Imagen. (2010). The IMAGEN study: reinforcement-related behaviour in normal brain function and psychopathology. *Molecular Psychiatry, 15*(12), 1128-1139. doi:10.1038/mp.2010.4

Stefanis, N. C., Hanssen, M., Smirnis, N. K., Avramopoulos, D. A., Evdokimidis, I. K., Stefanis, C. N., . . . Van Os, J. (2002). Evidence that three dimensions of psychosis have a distribution in the general population. *Psychological Medicine, 32*(2), 347-358. doi:10.1017/s0033291701005141

Troyanskaya, O., Cantor, M., Sherlock, G., Brown, P., Hastie, T., Tibshirani, R., . . . Altman, R. B. (2001). Missing value estimation methods for DNA microarrays. *Bioinformatics, 17*(6), 520-525. doi:10.1093/bioinformatics/17.6.520

Trubetskoy, V., Pardinas, A. F., Qi, T., Panagiotaropoulou, G., Awasthi, S., Bigdeli, T. B., . . . Schizophrenia Working Group of the Psychiatric Genomics, Consortium. (2022). Mapping genomic loci implicates genes and synaptic biology in schizophrenia. *Nature, 604*(7906), 502-508. doi:10.1038/s41586-022-04434-5

Vapnik, V. N. (1999). An overview of statistical learning theory. *IEEE Transactions on Neural Networks, 10*(5), 988-999. doi:10.1109/72.788640

Varma, Sudhir, & Simon, Richard %J BMC bioinformatics. (2006). Bias in error estimation when using cross-validation for model selection. *BMC Bioinformatics, 7*(1), 1-8.

Walls, Theodore A., & Wielt, Dustin B. (2007). Bollen, K.A., & Curran, P.J. (2006). Latent curve models: A structural equation approach. Hoboken, NJ: Wiley. 285+xii pp. US$99.95. ISBN: 047145592X. *Psychometrika, 72*(4), 643-646. doi:10.1007/s11336-007-9011-6

Woicik, P. A., Stewart, S. H., Pihl, R. O., & Conrod, P. J. (2009). The Substance Use Risk Profile Scale: a scale measuring traits linked to reinforcement-specific substance use profiles. *Addictive behaviors, 34*(12), 1042-1055. doi:10.1016/j.addbeh.2009.07.001

Yu, Y., Shen, M., Niu, L., Liu, Y. E., Xiao, S., & Tebes, J. K. (2022). The relationship between clinical recovery and personal recovery among people living with schizophrenia: A serial mediation model and the role of disability and quality of life. *Schizophrenia Research, 239*, 168-175. doi:10.1016/j.schres.2021.11.043

Zheng, X., Levine, D., Shen, J., Gogarten, S. M., Laurie, C., & Weir, B. S. (2012). A high-performance computing toolset for relatedness and principal component analysis of SNP data. *Bioinformatics, 28*(24), 3326-3328. doi:10.1093/bioinformatics/bts606

**SUPPLEMENTARY TABLES**

**Supplementary Table 1.** Overview of the instruments collected within the IMAGEN study and employed for analysis purposes within this study.

| **Domains of interest** | **Total N of selected scores**  **(considered TP of collection)** | **Questionnaires** | **Assessed domains** | **Selected scores** |
| --- | --- | --- | --- | --- |
| **Personality, temperament and character** | 14  (BL, FU1, FU2) | **NEO Five-Factor Inventory (NEO-FFI)** | *Personality* | Openness  Conscentiousness  Extraversion  Agreeableness  Neuroticism |
|  |  |  |  |  |
|  |  | **Temperament and Character Inventory-Revised**  **(TCI-R)** | *Temperament and character* | Exploratory excitability  Impulsiveness  Extravagance  Disorderliness  Novelty Seeking |
|  |  | **Substance Use Risk Profile Scale (SURPS)** | *Personality risk factors for addictive behaviors* | Anxiety sensitivity  Hopelessness  Impulsivity  Sensation seeking |
| **Emotional and behavioral disorders** | 4  (FU3) | **Strength and Difficulties Questionnaire** administered via DAWBA package | *Estimated risk for emotional and behavioral disorders* | Risk for emotional disorders  Risk for conduct disorders  Risk for hyperactivity disorders  Risk for any other disorders |
| **Bullying victimization** | 1  (BL, FU1, FU2) | **Bully Questionnaire** | *Bullying victimization* | Victimization |
|  |  |  |  |  |

**Abbreviations:** BL=Baseline; FU1=Follow-up 1; FU2=Follow-up 2; FU3=Follow-up 3; TP=Time Points.

**Supplementary Table 2.** Mean and standard deviation values with between-group comparisons at Baseline, Follow-Up 1, and Follow-Up 2 for each selected raw measure of assessment (i.e., tool-specific personality, temperament and character scores, and Strenght and Difficulties Questionnaire scores of estimated risk derived from the Development and Well-Being Assessment package) in both Discovery and Validation samples.

| **Measures**  **of assessment** | Discovery  sample  (DS)  (mean±SD) | Validation  sample  (VS)  (mean±SD) | **DS vs. VS**  **T (p-value)** | DS  Higher-PPS  (mean±SD) | DS  Lower-PPS  (mean±SD) | **DS**  **Higher-PPS**  **vs.**  **Lower-PPS**  **T (uncorr. p) (FDR-corr. p)** | | VS  Higher-PPS  (mean±SD) | VS  Lower-PPS  (mean±SD) | **VS**  **Higher-PPS**  **vs.**  **Lower-PPS**  **T (uncorr. p)** |
| --- | --- | --- | --- | --- | --- | --- | --- | --- | --- | --- |
| *NEO neuroticism*  *BL*  *FU1*  *FU2* | 1.92±0.64  1.84±0.62  1.72±0.57 | 1.93±0.59  1.84±0.62  1.72±0.57 | -0.21(0*.84*)  -1.06(0*.29*)  -1.75(0*.08*) | 2.08±0.64  2.05±0.62  1.95±0.67 | 1.77±0.60  1.65±0.57  1.49±0.59 | 5.71(<0.001)*  7.76(<0.001)*  8.43(<0.001)* | (0.003)**  (0.003)**  (0.003)** | 1.90±0.60  1.90±0.71  1.85±0.77 | 1.97±0.59  1.90±0.62  1.77±0.65 | -0.98(0.33)  -0.06(0.95)  0.89(0.38) |
| *NEO extraversion*  *BL*  *FU1*  *FU2* | 2.51±0.49  2.47±0.51  2.49±0.49 | 2.46±0.49  2.42±0.49  2.44±0.48 | 1.33(0*.18*)  1.42(0*.15*)  1.17(0*.24*) | 2.44±0.51  2.39±0.51  2.37±0.51 | 2.58±0.45  2.56±0.48  2.61±0.45 | -3.36(0.001)*  -3.95(<0.001)*  -5.66(<0.001)* | (0.001)*  (0.003)**  (0.003)** | 2.46±0.46  2.44±0.47  2.43±0.49 | 2.47±0.53  2.39±0.51  2.47±0.46 | -0.10(0.92)  0.92(0.36)  -0.68(0.49) |
| *NEO openness*  *BL*  *FU1*  *FU2* | 2.25±0.50  2.32±0.49  1.92±0.52 | 2.22±0.48  2.35±0.47  2.42±0.53 | 0.90(0*.37*)  -0.57(0*.57*)  0.24(0*.81*) | 2.31±0.47  2.40±0.47  2.51±0.49 | 2.20±0.52  2.25±0.52  2.35±0.54 | 2.72(0.007)*  3.44(0.001)*  3.55(<0.001)* | (0.017)*  (0.003)**  (0.003)** | 2.19±0.50  2.31±0.47  2.38±0.53 | 2.26±0.44  2.39±0.47  2.47±0.53 | -1.18(0.24)  -1.30(0.19)  -1.34(0.18) |
| *NEO agreableness*  *BL*  *FU1*  *FU2* | 2.46±0.43  2.51±0.46  2.67±0.48 | 2.45±0.43  2.53±0.40  2.65±0.45 | 0.38(0*.70*)  -0.48(0*.63*)  0.77(0*.44*) | 2.44±0.44  2.46±0.47  2.65±0.48 | 2.48±0.42  2.57±0.45  2.70±0.48 | -0.91(0.36)  -2.67(0.008)*  -1.29(0.19) | (0.43)  (0.019)*  (0.26) | 2.44±0.45  2.53±0.41  2.66±0.45 | 2.46±0.41  2.53±0.40  2.63±0.45 | -0.29(0.77)  -0.12(0.90)  0.66(0.51) |
| *NEO consciousness*  *BL*  *FU1*  *FU2* | 2.32±0.57  2.35±0.59  2.53±0.60 | 2.38±0.55  2.39±0.54  2.49±0.59 | -1.23(0*.22*)  -0.84(0*.40*)  1.03(0*.30*) | 2.28±0.49  2.28±0.58  2.47±0.59 | 2.37±0.62  2.43±0.59  2.59±0.60 | -1.19(0.05)  -2.84(0.005)*  -2.34(0.02)* | (0.09)  (0.013)*  (0.02)* | 2.35±0.59  2.39±0.53  2.49±0.60 | 2.41±0.51  2.40±0.57  2.48±0.57 | -0.81(0.42)  -0.15(0.88)  0.16(0.88) |
| *TCI exploratory*  *excitability*  *BL*  *FU1*  *FU2* | 34.43±3.94  33.62±4.19  33.31±4.33 | 34.49±4.12  33.52±4.21  33.21±4.24 | -0.19(0*.85*)  0.32(0*.74*)  0.31(0*.76*) | 34.27±3,81  33.40±4.36  33.03±4.61 | 34.59±4.07  33.85±4.02  33.59±4.03 | -0.93(0.35)  -1.22(0.22)  -1.49(0.14) | (0.43)  (0.29)  (0.20) | 34.25±3.99  33.38±4.07  32.84±4.17 | 34.79±4.26  33.70±4.38  33.67±4.30 | -1.06(0.29)  0.60(0.55)  -1.57(0.12) |
| *TCI impulsiveness*  *BL*  *FU1*  *FU2* | 25.65±4.34  25.59±4.02  24.95±3.79 | 25.94±4.10  25.24±3.66  24.99±3.98 | -0.89(0*.37*)  1.82(0.*24*)  -0.12(0*.91*) | 25.05±4.28  25.24±3.88  24.59±3.92 | 26.24±4.34  25.94±4.13  25.31±3.62 | -3.16(0.002)*  -2.01(0.04)*  -2.19(0.03)* | (0.006)**  (0.07)  (0.1) | 25.96±3.70  25.17±3.49  24.96±4.12 | 25.92±4.56  25.34±3.88  25.03±3.83 | 0.07(0.95)  -0.36(0.72)  -0.14(0.89) |
| *TCI extravagance*  *BL*  *FU1*  *FU2* | 28.07±4.27  27.77±4.01  27.53±4.42 | 27.89±4.27  27.71±3.89  27.68±4.13 | 0.57(0*.57*)  0.22(0*.83*)  -0.50(0*.62*) | 28.03±4.46  27.98±3.97  27.26±4.41 | 28.12±4.08  27.57±4.04  27.43±4.44 | -0.24(0.81)  1.19(0.24)  0.48(0.63) | (0.81)  (0.30)  (0.69) | 27.94±3.67  27.75±3.48  27.87±4.009 | 27.84±4.94  27.66±4.36  27.47±4.28 | 0.18(0.86)  0.17(0.87)  0.79(0.43) |
| *TCI disorderliness*  *BL*  *FU1*  *FU2* | 22.49±3.70  22.09±3.74  21.42±3.77 | 22.12±3.64  21.84±3.84  21.45±3.76 | 1.32(0.*19*)  0.91(0*.36*)  -0.01(0*.91*) | 22.16±3.57  22.24±3.79  21.18±3.73 | 22.82±3.8  21.96±3.69  21.66±3.79 | -2.02(0.04)*  0.84(0.39)  -1.49(0.14) | (0.07)  (0.45)  (0.20) | 22.07±3.71  22.17±3.66  21.37±3.65 | 22.19±3.56  21.43±4.02  21.56±3.91 | -0.26(0.79)  1.54(0.12)  -0.41(0.68) |
| *TCI novelty seeking*  *BL*  *FU1*  *FU2* | 110.65±10.48  109.07±10.49  107.22±10.45 | 110.45±10.27  108.30±10.10  107.34±10.23 | 0.26(0.80)  0.98(0*.33*)  -0.16(0*.87*) | 109.52±10.26  108.87±10.49  106.42±10.84 | 111.77±10.59  109.27±10.51  108±10.002 | -2,47(0.01)*  -0.43(0.66)  -1.74(0.08) | (0.04)*  (0.69)  (0.13) | 110.21±9.01  108.44±8.82  107.04±9.89 | 110.74±11.66  108.13±11.51  107.72±10.66 | -0.41(0.68)  0.24(0.80)  -0.54(0.59) |
| *SURPS anxiety*  *sensitivity*  *BL*  *FU1*  *FU2* | 11.38±2.26  11.24±2.14  11.74±2.38 | 11.27±2.25  11.31±2.29  11.93±2.54 | 0.64(0*.52*)  -0.40(0*.69*)  -1.01(0*.31*) | 11.70±2.36  11.54±2.15  12.07±2.29 | 11.06±2.10  10.94±2.09  11.42±2.43 | 3.30(0.001)*  3.27(0.001)*  3.14(0.002)* | (0.003)**  (0.003)**  (0.006)** | 11.02±2.17  11.16±2.15  11.70±2.58 | 11.57±2.31  11.48±2.44  12.21±2.47 | -1.96(0.05)  -1.12(0.26)  -1.61(0.11) |
| *SURPS hopelessness*  *BL*  *FU1*  *FU2* | 13.04±2.67  12.93±2.89  12.94±3.21 | 12.89±2.54  12.82±2.92  13.17±3.25 | 0.73(0.*46*)  0.53(0*.59*)  -0.92(0*.35*) | 13.58±2.73  13.51±2.92  13.79±3.38 | 12.51±2.50  12.38±2.75  12.11±2.79 | 4.67(<0.001)*  4.55(<0.001)*  6.21(<0.001)* | (0.003)**  (0.003)**  (0.003)** | 12.82±2.80  13.45±3.31  11.02±2.17 | 12.83±3.06  12.83±3.15  11.57±2.31 | 0.09(0.93)  -0.03(0.98)  1.53(0.126) |
| *SURPS impulsivity*  *BL*  *FU1*  *FU2* | 11.84±2.06  11.13±2.14  10.79±2.09 | 11.98±2.24  11.19±1.96  10.69±2.03 | -0.82(0*.41*)  -0.38(0*.70*)  -1.06(0*.29*) | 11.89±2.02  11.26±2.12  10.86±1.96 | 11.81±2.10  11.01±2.17  10.73±2.21 | 0.41(0.68)  1.31(0.19)  0.69(0.49) | (0.68)  (0.26)  (0.55) | 12.05±2.83  11.01±1.79  10.85±1.99 | 11.90±2.19  11.42±2.14  11.09±2.09 | 0.54(0.58)  -1.69(0.91)  0.95(0.34) |
| *SURPS sensation*  *seeking*  *BL*  *FU1*  *FU2* | 16.12±3.02  16.09±2.88  16.20±3.01 | 15.86±2.84  16.02±2.98  16.16±3.36 | 1.15(0*.25*)  0.31(0*.76*)  0.16(0*.87*) | 15.91±3.16  15.85±2.88  15.95±3.08 | 16.33±2.86  16.32±2.87  16.45±3.07 | -1.59(0.11)  -1.88(0.06)  -1.89(0.59) | (0.17)  (0.06)  (0.65) | 15.73±2.72  15.76±2.83  15.83±3.38 | 16.03±2.99  16.34±3.13  16.57±3.30 | -0.82(0.41)  -1.55(0.12)  -1.76(0.08) |
| *D-SDQ risk for emotional disorders*  *FU3* | 0.15±0.49 | 0.16±0.52 | -0.28(0*.78*) | 0.18±0.53 | 0.13±0.45 | 1.12(0.26) | n.a. | 0.30±0.67 | 0 | 5.34(0.0012)** |
| *D-SDQ risk for conduct disorders risk*  *FU3* | 0.02±0.18 | 0.01±0.13 | 0.75(0.*45*) | 0.02±0.12 | 0.03±0.22 | -1.20(0.23) | n.a. | 0.01±0.12 | 0.02±0.13 | -0.20(0.84) |
| *D-SDQ risk for hyperactivity disorders*  *FU3* | 0.07±0.25 | 0.08±0.27 | -0.56(0*.57*) | 0.05±0.23 | 0.08±0.27 | -1.18(0.24) | n.a. | 0.14±0.35 | 0 | 4.81(0.0012)* |
| *D-SDQ risk for any other disorder*  *FU3* | 0.20±0.53 | 0.22±0.55 | -0.38(0*.70*) | 0.21±0.55 | 0.20±0.52 | 0.23(0.82) | n.a. | 0.39±0.69 | 0.02±0.13 | 6.23(0.0012)* |
|  |  |  |  |  |  |  |  |  |  |  |

**Abbreviations**: BL=Baseline; corr.=corrected; D-SDQ= Strenght and Difficulties Questionnaire scores of estimated risk derived from the Development and Well-Being Assessment package; FU1=Follow-Up 1; FU2=Follow-Up 2; FU3=Follow-Up 3; NEO=NEO Five Factor Inventory; SD=Standard Deviation; SURPS=Substance Use Risk Profile Scale; TCI=Temperament and Character Inventory; uncorr.=uncorrected.

(*) marks p<0.05, (**) marks p<0.01, (***) marks p<0.001.

**Supplementary Table 3.** Demographic and clinical characteristics of the 653 individuals with available genetic data.

|  | All subjects (mean±SD) |
| --- | --- |
| *N* | 653 |
| *Gender Ratio (M/F)* | 303/350 |
| *Age in years*  *BL*  *FU1*  *FU2* | 13.9±0.4  16.007±0.4  18.4±0.6 |
| *CAPE-42 total score* | 85.1±25.8 |

**Abbreviations**: BL=Baseline; CAPE-42=Community Assessment of Psychic Experiences-42; FU1=Follow-Up 1; FU2=Follow-up 2; M/F=Male/Female; SD=Standard Deviation.

**Supplementary Table 4.** Mean and standard deviation values at Baseline, Follow-Up 1 and Follow-Up 2 of victimization total scores (as measured by Bully Questionnaire) of the 653 individuals with available genetic data.

|  | All subjects (mean±SD) |
| --- | --- |
| *BQ Victimization score*  *BL*  *FU1*  *FU2* | 7.9±2.8  7.2±1.8  6.7±1.8 |

**Abbreviations**: BL=Baseline; BQ=Bully Questionnaire; FU1=Follow-Up 1; FU2=Follow-Up 2; SD=Standard Deviation.

**Supplementary Table 5**. Goodness-of-fit indices of the Latent Growth Curve Models implemented for each selected personality variable in both the Discovery and Validation samples.

| Variable | Sample | χ2, p-value | CFI | TLI | RMSEA  (boostrapped 95% CI) | | SRMR |
| --- | --- | --- | --- | --- | --- | --- | --- |
| *NEO neuroticism* | **Discovery sample** | 8.95, p=0.25 | 0.996 | 0.994 | 0.023 (0-0.061) | 0.029 | |
|  | *Validation sample* | *9.71, p=0.20* | *0.994* | *0.990* | *0.027(0-0.064)* | *0.037* | |
| *NEO extraversion* | **Discovery sample** | 3.71, p=0.81 | 1 | 1.009 | 0 (0-0.033) | 0.014 | |
|  | *Validation sample* | *4.24, p=0.75* | *1* | *1.009* | *0 (0-0.038)* | *0.015* | |
| *NEO openness* | **Discovery sample** | 10.88, p=0.14 | 0.994 | 0.990 | 0.032 (0-0.068) | 0.036 | |
|  | *Validation sample* | *4.81, p=0.68* | *1* | *1.005* | *0 (0-0.042)* | *0.024* | |
| *NEO agreeableness* | **Discovery sample** | 10.14, p=0.18 | 0.995 | 0.991 | 0.029 (0-0.065) | 0.029 | |
|  | *Validation sample* | *16.24, p=0.02* | *0.983* | *0.971* | *0.050 (0.017-0.082)* | *0.041* | |
| *NEO consciousness* | **Discovery sample** | 2.28, p=0.94 | 1 | 1.012 | 0 (0-0.009) | 0.013 | |
|  | *Validation sample* | *1.33, p=0.99* | *1* | *1.016* | *0* | *0.009* | |
| *TCI exploratory excitability* | **Discovery sample** | 2.77, p=0.90 | 1 | 1.021 | 0 (0-0.219) | 0.010 | |
|  | *Validation sample* | *4.60, p=0.70* | *1* | *1.012* | *0 (0-0.040)* | *0.015* | |
| *TCI impulsiveness* | **Discovery sample** | 4.72, p=0.69 | 1 | 1.011 | 0 (0-0.041) | 0.021 | |
|  | *Validation sample* | *6.45, p=0.48* | *1* | *1.003* | *0 (0-0.051)* | *0.020* | |
| *TCI extravagance* | **Discovery sample** | 15.59, p=0.03 | 0.978 | 0.963 | 0.048 (0.014-0.080) | 0.030 | |
|  | *Validation sample* | *3.22, p=0.86* | *1* | *1.022* | *0 (0-0.028)* | *0.014* | |
| *TCI disorderliness* | **Discovery sample** | 17.31, p=0.01 | 0.969 | 0.948 | 0.052 (0.021-0.084) | 0.036 | |
|  | *Validation sample* | *7.94, p=0.34* | *0.997* | *0.995* | *0.016 (0-0.057)* | *0.025* | |
| *TCI novelty seeking* | **Discovery sample** | 7.82, p=0.35 | 0.998 | 0.997 | 0.015 (0-0.057) | 0.021 | |
|  | *Validation sample* | *4.36, p=0.74* | *1* | *1.010* | *0 (0-0.039)* | *0.018* | |
| *SURPS anxiety sensitivity* | **Discovery sample** | 3.64, p=0.82 | 1 | 1.021 | 0 (0-0.032) | 0.011 | |
|  | *Validation sample* | *4.96, p=0.66* | *1* | *1.013* | *0 (0-0.043)* | *0.019* | |
| *SURPS hopelessness* | **Discovery sample** | 5.71, p=0.57 | 1 | 1.007 | 0 (0-0.047) | 0.027 | |
|  | *Validation sample* | *11.16, p=0.13* | *0.983* | *0.971* | *0.033 (0-0.069)* | *0.037* | |
| *SURPS impulsivity* | **Discovery sample** | 16.79, p=0.02 | 0.971 | 0.951 | 0.051 (0.019-0.083) | 0.038 | |
|  | *Validation sample* | *12.57, p=0.08* | *0.981* | *0.968* | *0.038 (0-0.073)* | *0.035* | |
| *SURPS sensation seeking* | **Discovery sample** | 10.12, p=0.18 | 0.994 | 0.990 | 0.029 (0-0.065) | 0.029 | |
|  | *Validation sample* | *10.20, p=0.17* | *0.994* | *0.990* | *0.029 (0-0.066)* | *0.034* | |

**Abbreviations**: CFI=Comparative Fit Index; CI=Confidence Interval; NEO=NEO Five Factor Inventory; RMSEA=Root Mean Square Error of Approximation; SRMR=Standardized Root Mean Square Residual; SURPS=Substance Use Risk Profile Scale; TCI=Temperament and Character Inventory; TLI=Tucker-Lewis Index.

**Supplementary Table 6.** Mean and standard deviation values for each cognitive variable correlated with Higher vs. Lower Psychosis Proneness Signs prediction scores in the Discovery sample.

|  | Discovery sample (mean±SD) |
| --- | --- |
| BL PRM - correct responses (%) | 94.55±8.40 |
| BL RVP – accuracy | 0.89±0.05 |
| BL SWM - between loads error score | 18.71±13.57 |
| BL SWM - strategy score | 31.03±5,29 |
| BL AGN – correct answers to positive stimuli: mean latency | 474.51±106.01 |
| BL AGN – correct answers to negative stimuli: mean latency | 490.17±113.09 |
| BL AGN – number of total omissions for positive stimuli | 12.99±7.08 |
| BL AGN – number of total omissions for negative stimuli | 11.32±7.74 |
| BL CGT – delay aversion score | 0.24±0.14 |
| BL CGT – deliberation time (ms) | 2002.67±663.13 |
| BL CGT – overall proportion bet score | 0.47±0.12 |
| BL CGT – quality of decision making score | 0.94±0.08 |
| BL CGT – risk adjustment score | 1.68±1 |
| BL CGT – risk taking score | 0.52±0.13 |
| BL PALP – PP condition score | 310.70±56.62 |
| BL PALP – RP condition score | 252.18±49.01 |
| BL PALP – RR condition score | 360.56±61.13 |
| BL PALP – mean omissions for PP condition | 0.04±0.05 |
| BL PALP – mean omissions for RP condition | 0.05±0.07 |
| BL PALP – mean omissions for RR condition | 0.05±0.06 |
| BL PALP – mean omissions for PP condition | 0.13±0.11 |
| BL PALP – mean omissions for RP condition | 0.12±0.09 |
| BL PALP – mean omissions for RR condition | 0.15±0.12 |
| BL PALP – reaction times for PP condition (ms) | 1006.06±269.16 |
| BL PALP – reaction times for RP condition (ms) | 936.47±220.67 |
| BL PALP – reaction times for RR condition (ms) | 1006±296 |

**Abbreviations**: AGN=Affective Go-No-Go; BL=Baseline; CGT=Cambridge Guessing Task; ms=milliseconds; PALP=Passive Avoidance Learning Paradigm; PP=Punishment-Punishment; PRM=Pattern Recognition Memory task; RP=Reward-Punishment; RR=Reward-Reward; RVP=Rapid Visual Information Processing task; SWM=Spatial Working Memory task.

|  | Discovery sample (mean±SD) |
| --- | --- |
| BL AUDIT – alcohol frequency of use score | 0.91±1.27 |
| BL AUDIT – dependance symptoms scores | 0.11±0.43 |
| BL AUDIT – alcohol harmful use score | 0.17±0.72 |
| BL AUDIT – total score | 1.18±1.97 |
| BL FTND – total scores | 0.02±0.20 |
| BL ESPAD – amphetamine lifelong frequency of use score | 0.002±0.04 |
| BL ESPAD – anabolic lifelong frequency of use score | 0.002±0.04 |
| BL ESPAD – cocaine lifelong frequency of use score | 0.004±0.09 |
| BL ESPAD – crack lifelong frequency of use score | 0.002±0.04 |
| BL ESPAD – glue lifelong frequency of use score | 0.04±0.27 |
| BL ESPAD – hashish lifelong frequency of use score | 0.08±0.51 |
| BL ESPAD – LSD lifelong frequency of use score | 0.002±0.04 |
| BL ESPAD – MDMA lifelong frequency of use score | 0.002±0.04 |
| BL ESPAD – mushrooms lifelong frequency of use score | 0.008±0.14 |
| BL ESPAD – narcotic lifelong frequency of use score | 0.01±0.23 |
| BL ESPAD – tranquilizers lifelong frequency of use score | 0.01±0.17 |

**Supplementary Table 7**. Mean and standard deviation values for each substance use variable correlated with Higher vs. Lower Psychosis Proneness Signs prediction scores in the Discovery sample.

**Abbreviations**: AUDIT= Alcohol Use Disorders Identification Test; BL=Baseline; ESPAD=European School Survey Project on Alcohol and Other Drugs tool; FTND= Fagerstrom Test for Nicotine Dependence.

**Supplementary Table 8**. Correlation analyses between single-subject prediction scores for Psychotic Proneness Symptoms extracted from the Discovery Sample personality-based risk calculator and scores at Baseline for cognitive performance in the Discovery sample. Spearman’s rho coefficients and *(p-values)* are shown. All significant p values were <.05, False Discovery Rate (FDR) corrected.

|  | DS Higher-PPS  vs. Lower-PPS  prediction scores  rho (*p-value*) |
| --- | --- |
| BL PRM - correct responses (%) | 0.02 *(0.74)* |
| BL RVP – accuracy | 0.10 *(0.15)* |
| BL SWM - between loads error score | -0.05 *(0.55)* |
| BL SWM - strategy score | -0.04 *(0.55)* |
| BL AGN – correct answers to positive stimuli: mean latency | -0.04 *(0.55)* |
| BL AGN – correct answers to negative stimuli: mean latency | -0.04 *(0.55)* |
| BL AGN – number of total omissions for positive stimuli | 0.09*(0.18)* |
| BL AGN – number of total omissions for negative stimuli | 0.10 *(0.15)* |
| BL CGT – delay aversion score | -0.01 *(0.77)* |
| BL CGT – deliberation time (ms) | 0.04 *(0.55)* |
| BL CGT – overall proportion bet score | 0.08 *(0.22)* |
| BL CGT – quality of decision making score | -0.02 *(0.74)* |
| BL CGT – risk adjustment score | 0.08 *(0.23)* |
| BL CGT – risk taking score | 0.10 *(0.15)* |
| BL PALP – PP condition score | 0.04 *(0.55)* |
| BL PALP – RP condition score | 0.07 *(0.25)* |
| BL PALP – RR condition score | 0.05 *(0.55)* |
| BL PALP – mean omissions for PP condition | -0.02 *(0.55)* |
| BL PALP – mean omissions for RP condition | 0.01 *(0.15)* |
| BL PALP – mean omissions for RR condition | 0.02 *(0.25)* |
| BL PALP – mean omissions for PP condition | -0.04 *(0.76)* |
| BL PALP – mean omissions for RP condition | -0.11 *(0.76)* |
| BL PALP – mean omissions for RR condition | -0.08 *(0.75)* |
| BL PALP – reaction times for PP condition (ms) | 0.04 *(0.55)* |
| BL PALP – reaction times for RP condition (ms) | -0.01 *(0.76)* |
| BL PALP – reaction times for RR condition (ms) | 0.04 *(0.55)* |

**Abbreviations**: AGN=Affective Go-No-Go; BL=Baseline; CGT=Cambridge Guessing Task; DS=Discovery Sample; PALP=Passive Avoidance Learning Paradigm; PP=Punishment-Punishment; PPS=Psychotic Proneness Signs; PRM=Pattern Recognition Memory task; RP=Reward-Punishment; RR=Reward-Reward; RVP=Rapid Visual Information Processing task; SWM=Spatial Working Memory task.

**Supplementary Table 9**. Correlation analyses between single-subject prediction scores for Psychotic Proneness Symptoms extracted from the Discovery Sample personality-based risk calculator and scores at Baseline for substance use in the Discovery sample. Spearman’s rho coefficients and *(p-values)* are shown. All significant p values were <.05, False Discovery Rate (FDR) corrected.

|  | DS Higher-PPS  vs. Lower-PPS  prediction scores  rho (*p-value*) |
| --- | --- |
| BL AUDIT – alcohol frequency of use score | -0.06 *(0.67)* |
| BL AUDIT – dependance symptoms scores | -0.05 *(0.67)* |
| BL AUDIT – alcohol harmful use score | -0.05 *(0.67)* |
| BL AUDIT – total score | -0.07 *(0.67)* |
| BL FTND – total scores | 0.03 *(0.67)* |
| BL ESPAD – amphetamine lifelong frequency of use score | -0.05 *(0.67)* |
| BL ESPAD – anabolic lifelong frequency of use score | -0.001 *(0.97)* |
| BL ESPAD – cocaine lifelong frequency of use score | -0.04 *(0.67)* |
| BL ESPAD – crack lifelong frequency of use score | 0.03 *(0.67)* |
| BL ESPAD – glue lifelong frequency of use score | -0.06 *(0.67)* |
| BL ESPAD – hashish lifelong frequency of use score | -0.005 *(0.97)* |
| BL ESPAD – LSD lifelong frequency of use score | -0.04 *(0.67)* |
| BL ESPAD – MDMA lifelong frequency of use score | -0.009 *(0.97)* |
| BL ESPAD – mushrooms lifelong frequency of use score | -0.05 *(0.67)* |
| BL ESPAD – narcotic lifelong frequency of use score | -0.03 *(0.67)* |
| BL ESPAD – tranquilizers lifelong frequency of use score | 0.007 *(0.97)* |

**Abbreviations**: AUDIT= Alcohol Use Disorders Identification Test; BL=Baseline; ESPAD=European School Survey Project on Alcohol and Other Drugs tool; FTND= Fagerstrom Test for Nicotine Dependence.

**Supplementary Table 10.** Indirect effects of polygenic risk for schizophrenia on final Psychosis Proneness Signs severity levels, through predictions from our Machine Learning personality-based risk calculator and the rank product of Bullying Victimization scores collected at Baseline, Follow Up 1, and Follow Up 2.

|  | effect | bootstrapped se | bootstrapping 95% confidence interval | |
| --- | --- | --- | --- | --- |
|  |  |  | **Lower** | **Upper** |
| Total indirect effect | 0.0552* | 0.0183 | 0.0201 | 0.0926 |
| Polygenic risk for schizophrenia 🡪 Personality-based predictions 🡪 PPS severity levels at FU3 | 0.0523* | 0.0167 | 0.0213 | 0.0867 |
| Polygenic risk for schizophrenia 🡪 Bullying victimization 🡪 PPS severity levels at FU3 | -0.0016 | 0.0042 | -0.0112 | 0.0060 |
| PRS for schizophrenia 🡪 Personality-based predictions 🡪 Bullying victimization 🡪 PPS severity levels at FU3 | 0.0045* | 0.0025 | 0.0006 | 0.0105 |

**Abbreviations**: FU3=Follow-Up 3; PPS=Psychotic Proneness Signs.

* marks significant indirect effects.

**Supplementary Table 11.** Indirect effect of polygenic risk for schizophrenia on final Psychosis Proneness Signs (PPS) severity levels, through predictions from our Machine Learning personality-based risk calculator and moderated mediation effect of the rank product of Peer Victimization scores as collected at Baseline, Follow Up 1, and Follow Up 2 (i.e., Bullying victimization) on the personality-PPS pathway.

|  | indirect effect | bootstrapped se | bootstrapping 95% confidence interval | |
| --- | --- | --- | --- | --- |
|  |  |  | **Lower** | **Upper** |
| Polygenic risk for schizophrenia 🡪 Personality-based predictions 🡪 PPS severity levels at FU3 | 0.0523* | 0.0168 | 0.0213 | 0.0869 |
| Index of moderated mediation  (Personality-based predictions  x Bullying victimization) | -0.0002 | 0.0330 | -0.0649 | 0.0646 |
|  |  |  |  |  |

**Abbreviations**: FU3=Follow-Up 3; PPS=Psychotic Proneness Signs.

* marks significant indirect effects.

**Supplementary Table 12.** Indirect effect of polygenic risk for schizophrenia on final Psychosis Proneness Signs (PPS) severity levels, through predictions from our Machine Learning personality-based risk calculator and moderated mediation effect of the rank product of Peer Victimization scores as collected at Baseline, Follow Up 1, and Follow Up 2 (i.e., Bullying victimization) on the polygenic risk score-PPS pathway.

|  | indirect effect | bootstrapped se | bootstrapping 95% confidence interval | |
| --- | --- | --- | --- | --- |
|  |  |  | **Lower** | **Upper** |
| Polygenic risk for schizophrenia 🡪 Personality-based predictions 🡪 PPS severity levels at FU3 | 0.0522* | 0.0167 | 0.0212 | 0.0867 |
| Index of moderated mediation  (Polygenic risk for schizophrenia  x Bullying victimization) | 0.0397 | 0.0330 | -0.0250 | 0.1044 |

**Abbreviations**: FU3=Follow-Up 3; PPS=Psychotic Proneness Signs.

* marks significant indirect effects.

**Supplementary Table 13.** Overview of the instruments collected within the TRAILS study and employed for replication purposes within this study.

| **Domains of interest** | **Total N of selected scores** | **Questionnaires**  considered time points of collection | **Assessed domains** | **Informant** | **Selected scores** |
| --- | --- | --- | --- | --- | --- |
| **Personality, temperament and character** | 12 | **Revised NEO Personality Inventory (NEO-PI-R)**  Wave 3 | *Personality* | Children | Vulnerability to stress  Excitement seeking  Impulsivity  Assertiveness  Self-discipline  Hostility |
|  |  |  |  |  |  |
|  |  | **Early Adolescence Temperament questionnire**  **(EATq)**  Wave 1, Wave 3 | *Temperament and character* | Parents | Affiliation  Effortful control  Fearfilness  Frunstration  Shyness  Surgency |
| **Bullying victimization** | 2 | **Youth Self-Report (YSR)**  Wave 2, Wave 3  **Child Behavior Checklist (CBCL)**  Wave 2, Wave 3 | *Bullying victimization* | Children  Parents | Item 38: “I get teased”  Item 38 “Get teased” |
|  |  |  |  |  |  |

**Supplementary Table 14.** Mean and standard deviation values with between-group comparisons at Wave 1 and Wave 3 for each selected raw measure of assessment (i.e., tool-specific personality, temperament and character scores) in the 1,546 individuals included in the external TRAILS replication cohort.

| **Measures**  **of assessment** | **All subjects**  **(mean±SD)** | **Higher-PPS**  **(mean±SD)** | **Lower-PPS**  **(mean±SD)** | **Higher-PPS**  **vs. Lower-PPS**  **T (p-value)** |
| --- | --- | --- | --- | --- |
| *NEO Vulnerability to stress*  *Wave 3* | 0.0008±0.99 | 0.20±1.06 | -0.19±0.90 | -7.75(<0.001***) |
| *NEO Excitement seeking*  *Wave 3* | 0.0009±0.99 | -0.04±1.04 | 0.04±0.95 | 1.59(0.11) |
| *NEO Impulsivity*  *Wave 3* | -0.00005±0.99 | 0.20±0.99 | -0.19±0.97 | -7.77(<0.001***) |
| *NEO Assertiveness*  *Wave 3* | 0.00006±0.99 | -0.09±1.02 | 0.08±0.97 | 3.35(0.001***) |
| *NEO Self-discipline*  *Wave 3* | -0.0005±0.99 | -0.13±1.02 | 0.12±0.96 | 5.07(<0.001***) |
| *NEO Hostility*  *Wave 3* | 0.0004±0.99 | 0.2±1 | -0.21±0.95 | -8.76(<0.001***) |
| *EATq Affiliation*  *Wave 1*  *Wave 3* | 0.0003±0.96  0.002±0.93 | 0.03±0.97  0.01±0.91 | -0.03±0.95  -0.006±0.95 | -1.38(0.17)  -0.36(0.72) |
| *EATq Effortful control*  *Wave 1*  *Wave 3* | 0.005±0.97  0.003±0.94 | -0.04±0.96  0.01±0.93 | 0.05±0.98  0.005±0.95 | 1.84(0.66)  0.06(0.95) |
| *EATq Fearfulness*  *Wave 1*  *Wave 3* | -0.008±0.97  -0.008±0.93 | 0.10±0.99  0.11±0.94 | -0.11±0.93  -0.11±0.91 | -4.36(<0.001***)  -4.79(<0.001***) |
| *EATq Frustration*  *Wave 1*  *Wave 3* | -0.006±0.96  0.02±0.95 | 0.05±1.03  0.07±0.93 | -0.06±0.90  -0.02±0.96 | -2.31(0.02*)  -1.80(0.07) |
| *EATq Shyness*  *Wave 1*  *Wave 3* | 0.002±0.97  0.001±0.93 | 0.07±1  0.07±0.90 | -0.06±0.92  -0.06±0.95 | -2.71(0.007**)  -2.91(0.004**) |
| *EATq Surgency*  *Wave 1*  *Wave 3* | 0.006±0.96  0.003±0.93 | -0.03±0.99  -0.08±0.92 | 0.05±0.94  0.08±0.94 | 1.77(0.07)  3.51(<0.001***) |

**Abbreviations**: EATq=Early Adolescence Temperament questionnaire; NEO=Revised NEO Personality Inventory; SD=Standard Deviation.

**Supplementary Table 15.** Demographic and clinical characteristics of the 1,132 individuals with available genetic data.

|  | All subjects (mean±SD) |
| --- | --- |
| *N* | 1,132 |
| *Gender Ratio (M/F)* | 619/513 |
| *Age in years*  *Wave 2*  *Wave 3* | 13.49±0.66  16.19±0.65 |
| *CAPE-42 total score* | 2.88±0.53 |

**Abbreviations**: CAPE-42=Community Assessment of Psychic Experiences-42; M/F=Male/Female; SD=Standard Deviation.

**Supplementary Table 16.** Answer frequencies (N) to each item-38 Likert point from the children-reported Youth Self-Report and the parents-reported Child Behavior Checklist as collected at Wave 2 and Wave 3 for the 1,132 individuals with available genetic data.

| Answer N | | | | |
| --- | --- | --- | --- | --- |
|  | **Missing**  **data** | **Likert point: 0** | **Likert point: 1** | **Likert point: 2** |
| *Bullying victimization via YSR (item 38)*  *Wave 2*  *Wave 3* | 10  6 | 922  1,039 | 172  78 | 30  9 |
| *Bullying victimization via CBCL (item 38)*  *Wave 2*  *Wave 3* | 26  105 | 937  914 | 155  100 | 14  13 |

**Abbreviations**: CBCL=Child Behavior Checklist; YSR=Youth Self-Report.

**Supplementary Table 17.** Indirect effects of polygenic risk for schizophrenia on final Psychosis Proneness Signs severity levels, through predictions from our Machine Learning personality-based risk calculator and the rank product of children-reported Bullying Victimization scores collected via the Youth Self-Report scale at Waves 2 and 3.

|  | effect | bootstrapped se | bootstrapping 95% confidence interval | |
| --- | --- | --- | --- | --- |
|  |  |  | **Lower** | **Upper** |
| Total indirect effect | 0.0212* | 0.0102 | 0.0018 | 0.0418 |
| Polygenic risk for schizophrenia 🡪 Personality-based predictions 🡪 PPS severity levels at w3 | 0.0222* | 0.0087 | 0.0055 | 0.0400 |
| Polygenic risk for schizophrenia 🡪 children-reported Bullying victimization 🡪 PPS severity levels at w3 | -0.0030 | 0.0035 | -0.0106 | 0.0035 |
| PRS for schizophrenia 🡪 Personality-based predictions 🡪 children-reported Bullying victimization 🡪 PPS severity levels at w3 | 0.0020* | 0.0010 | 0.0004 | 0.0044 |

**Abbreviations**: PPS=Psychotic Proneness Signs; w3=wave 3.

* marks significant indirect effects.

**Supplementary Table 18.** Indirect effects of polygenic risk for schizophrenia on final Psychosis Proneness Signs severity levels, through predictions from our Machine Learning personality-based risk calculator and the rank product of parents-reported Bullying Victimization scores collected via the Child Behavior Checklist at Waves 2 and 3.

|  | effect | bootstrapped se | bootstrapping 95% confidence interval | |
| --- | --- | --- | --- | --- |
|  |  |  | **Lower** | **Upper** |
| Total indirect effect | 0.0262* | 0.0101 | 0.0076 | 0.0472 |
| Polygenic risk for schizophrenia 🡪 Personality-based predictions 🡪 PPS severity levels at w3 | 0.0228* | 0.0089 | 0.0063 | 0.0413 |
| Polygenic risk for schizophrenia 🡪 parents-reported Bullying victimization 🡪 PPS severity levels at w3 | 0.0020* | 0.0026 | -0.0029 | 0.0078 |
| PRS for schizophrenia 🡪 Personality-based predictions 🡪 parents-reported Bullying victimization 🡪 PPS severity levels at w3 | 0.0014* | 0.0008 | 0.0002 | 0.0035 |

**Abbreviations**: PPS=Psychotic Proneness Signs; w3=wave 3.

* marks significant indirect effects.

**Supplementary Table 19.** Validated performance comparison between the risk calculator based on non-cross-validated VS. cross-validated coefficients of change.

|  | Sample | Sensitivity | Specificity | Balanced Accuracy | Area Under the Curve | Positive Predictive Value | Negative Predictive Value |
| --- | --- | --- | --- | --- | --- | --- | --- |
| Non-cross-validated coefficients of change | *Discovery* | 70.2 | 60.9 | **65.6** | 0.71 | 64.6 | 66.8 |
|  | *Validation* | *73.2* | *65.5* | ***69.4*** | *0.72* | *63.4* | *75* |
| Cross-validated coefficients of change | *Discovery* | 72.1 | 60.9 | **66.5** | 0.70 | 65.2 | 68.2 |
|  | *Validation* | *70.7* | *62.7* | ***66.7*** | *0.69* | *60.7* | *72.3* |

**SUPPLEMENTARY FIGURES**

**Supplementary Figure 1.** Consort chart resuming details about the subsequent steps of participant retention across the study.


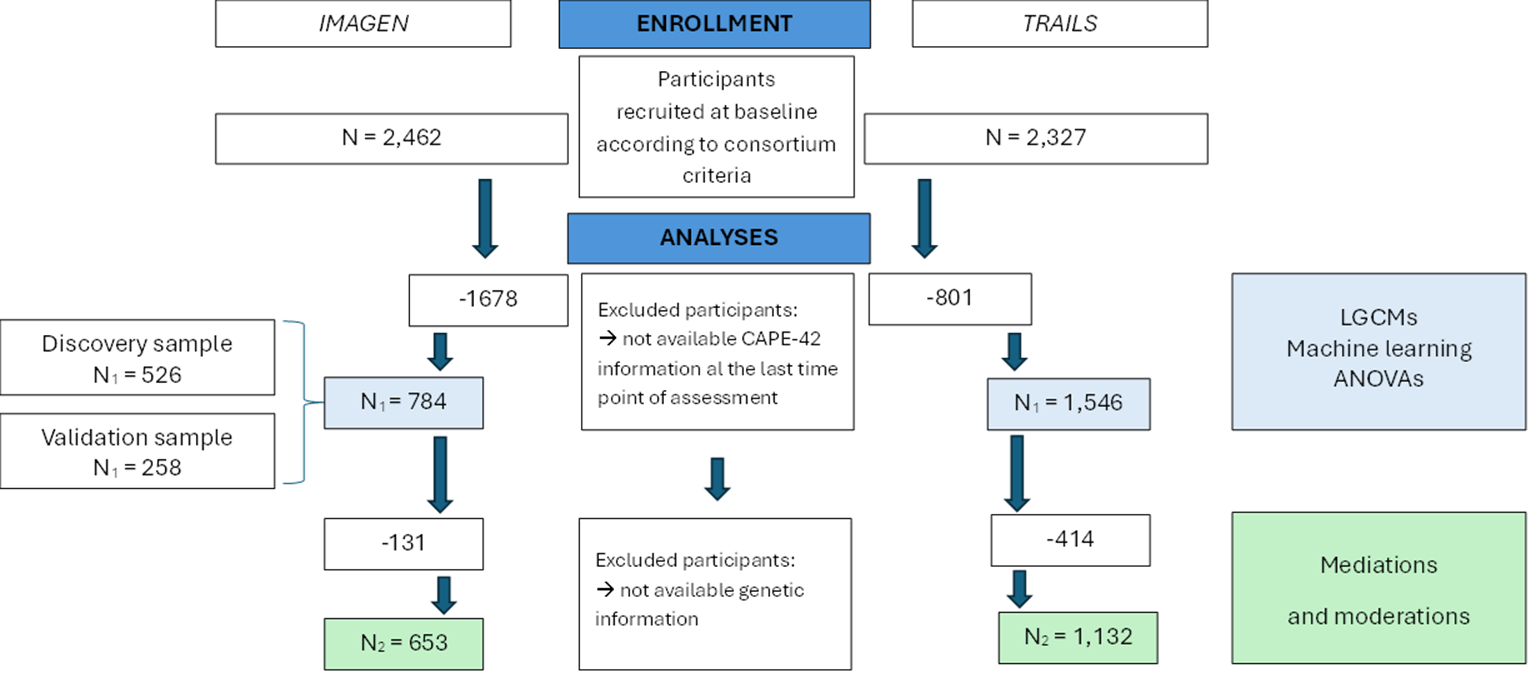


**Abbreviations**: CAPE-42= Community Assessment of Psychic Experiences-42; LGCMs=Latent Growth Curve Models; ANOVAs=ANalyses Of VAriance

**Supplementary Figure 2.** Depicting of the serial mediation (**2A**) and moderated mediation (**2B, 2C**) analyses designs. All the models included polygenic risk for schizophrenia as a predictor, Psychosis Proneness Signs severity levels at Follow-Up 3 (FU3) as a dependent variable, and personality-based predictions extracted from our Machine Learning personality-based risk calculator as first-order mediator. In the serial mediation model (2A), Bullying Victimization entered the model as second-order, serial mediator. In the moderated mediation models, Bullying Victimization entered the model as a moderator of the pathways between both personality-based predictions and polygenic risk for schizophrenia toward PPS at FU3 (2B and 2C respectively).


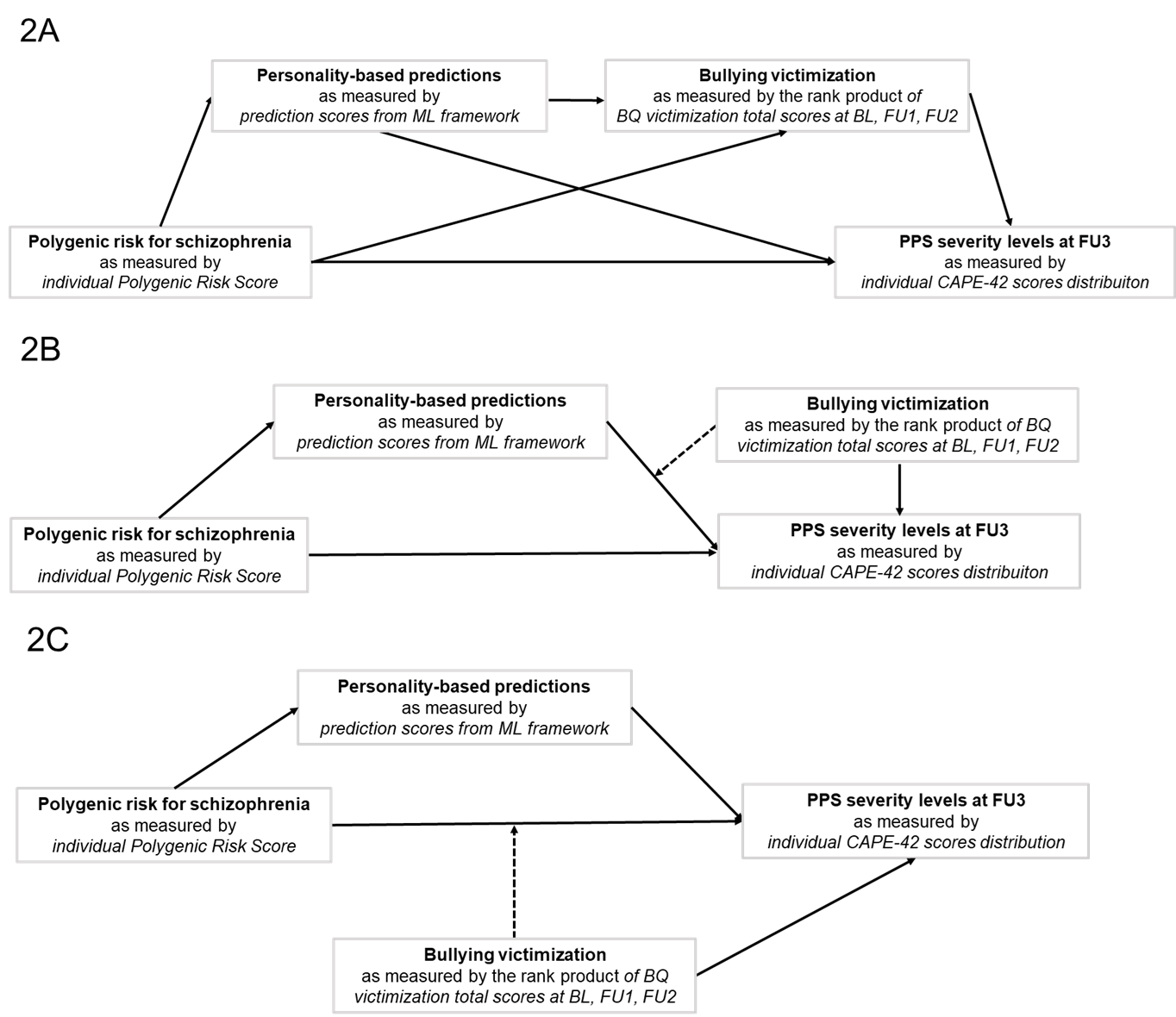


**Abbreviations**: BL=Baseline; BQ: Bully Questionnaire; CAPE-42=Community Assessment of Psychic Experiences-42; FU1/2/3=Follow-Up 1/2/3; ML=Machine Learning; PPS=Psychosis Proneness Signs.

**Supplementary Figure 3.** Probability of each feature for being selected in the Machine Learning Cross-Validation framework of our personality-based risk calculator in the Discovery Sample. Scores closer to 1 represent a higher probability of being selected for decision by the Support Vector Machine algorithm. Pink bars represent features with a selection probability > 0.5.


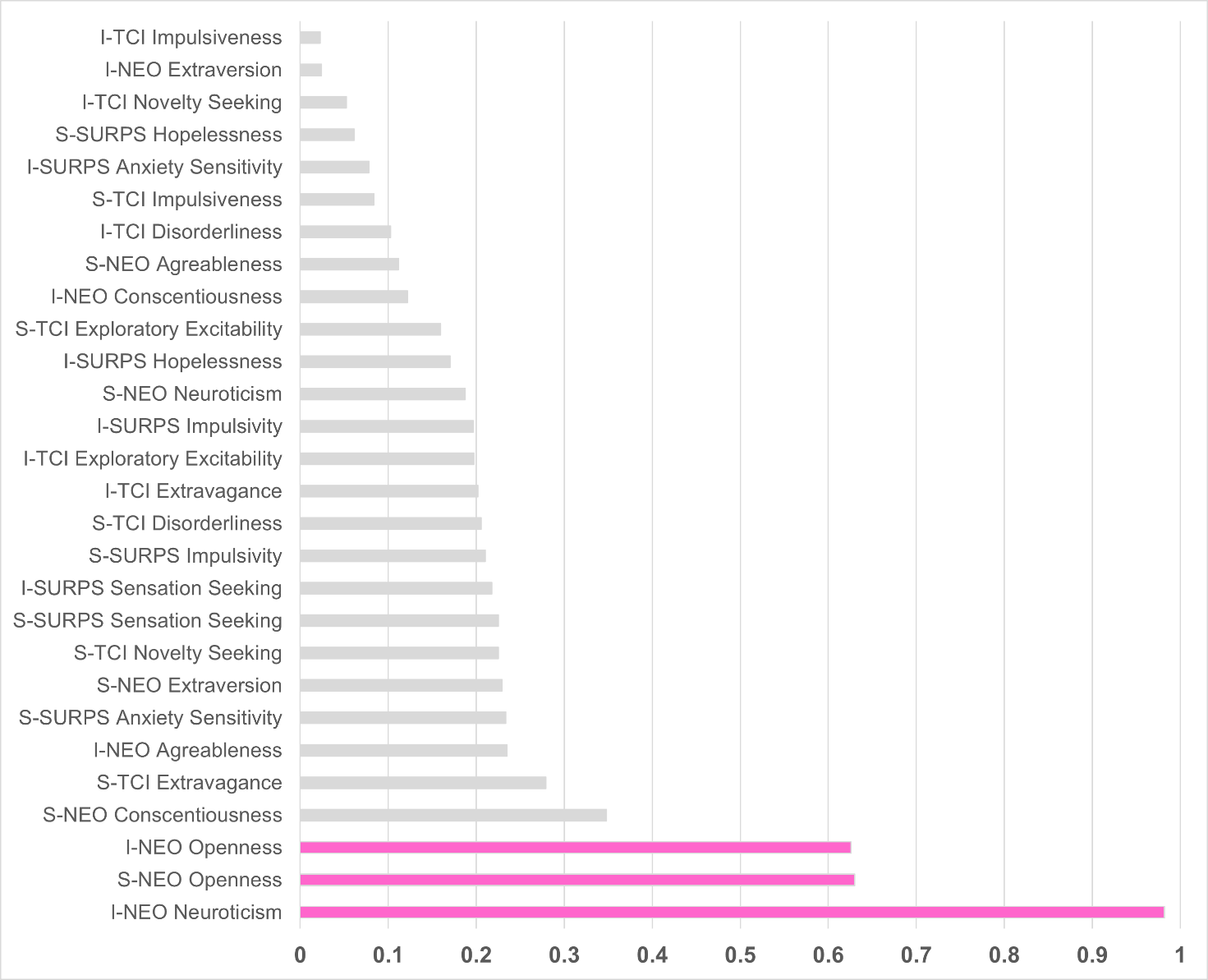


**Abbreviations**: I=intercept; NEO=NEO Five Factor Inventory; S=slope; SURPS=Substance Use Risk Profile Scale; TCI=Temperament and Character Inventory.


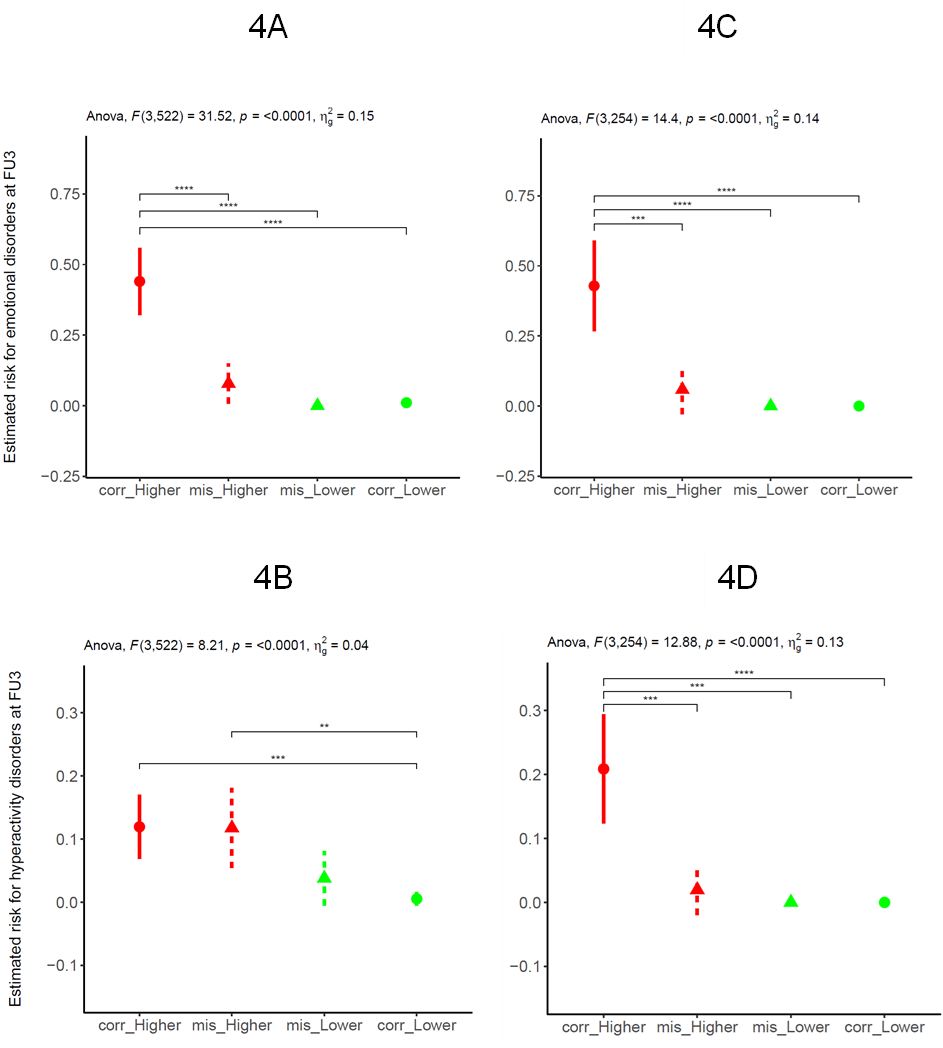
**Supplementary Figure 4.** Significant main effect of prediction rate categories extracted from the personality-based risk calculator on scores for emotional and hyperactivity disorders at Follow-Up 3 (estimated via the Development and Well-Being Assessment algorithm from the Strength and Difficulties Questionnaire) in both Discovery (left panels: 4A, 4B) and Validation (right panels: 4C, 4D) sample. Error bars represent standard error.

**Abbreviations**: corr=correctly predicted; mis=mispredicted; * marks p<0.05; ** marks p<0.01; *** marks p<0.001; **** marks p<0.0001

**Supplementary Figure 5**. Findings from the moderated mediation models, investigating the role of Bullying Victimization rank product in moderating the pathways toward Psychosis Proneness Signs from personality-based predictions (5A) and polygenic risk for schizophrenia (5B) respectively. Direct effects (unstandardized coefficients) are shown. Red arrows represent relationships returning significant direct effects. The dashed grey arrows represent a not-significant moderation effect. Indirect effects for each model are reported in Supplementary Tables 11 and 12.


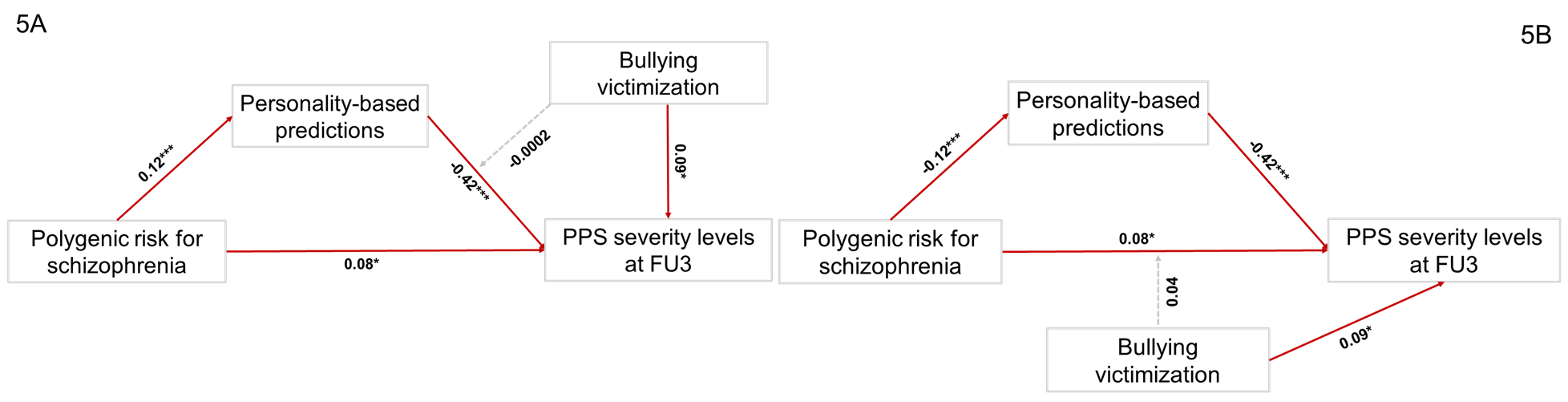


Abbreviations: FU3=Follow-Up 3; PPS=Psychosis Proneness Signs; * marks p<0.05; ** marks p<0.01; *** marks p<0.001.


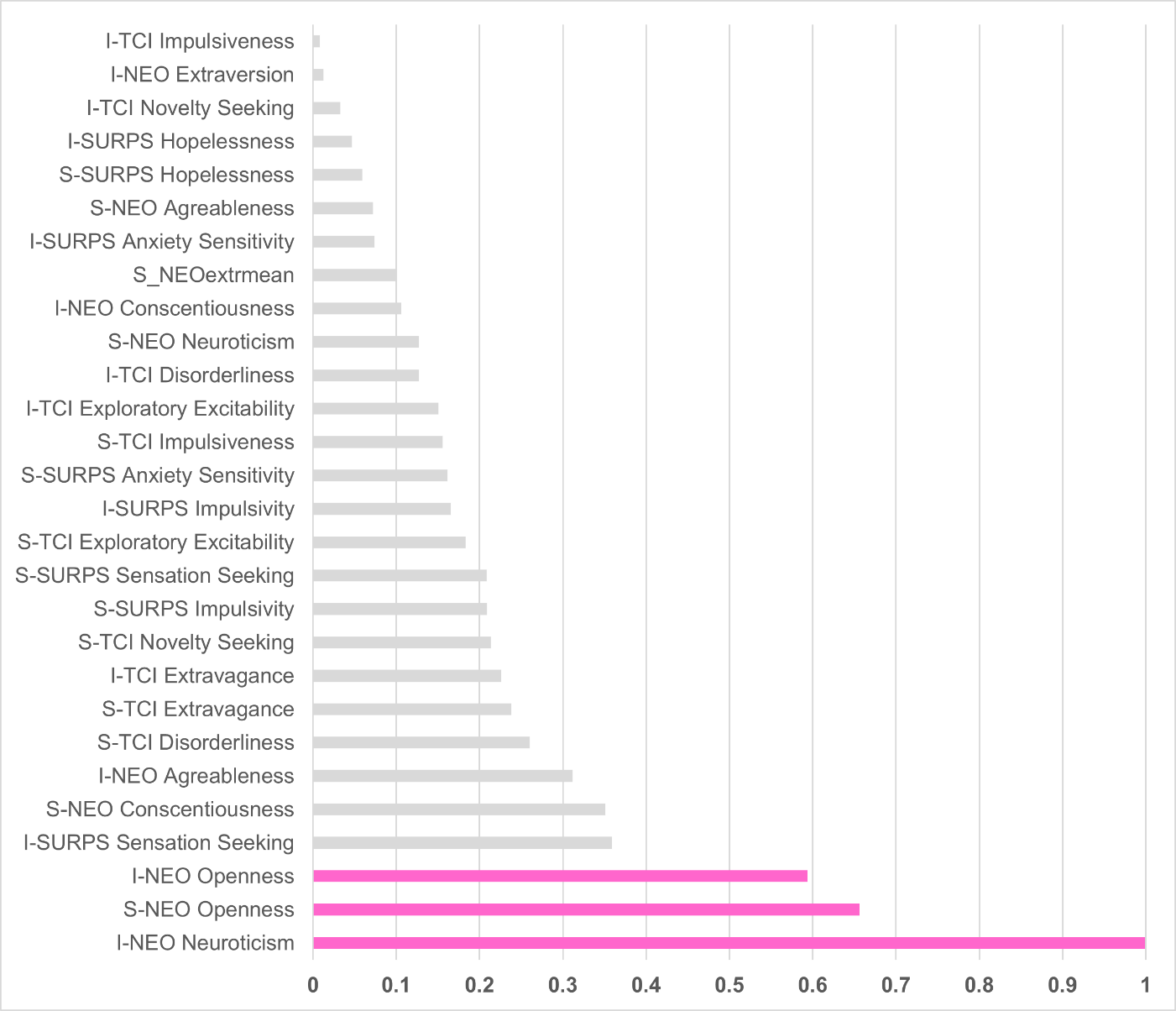
**Supplementary Figure 6.** Probability of each feature for being selected in the Machine Learning Cross-Validation framework for the risk calculator based on cross-validated coefficients of personality change. Scores closer to 1 represent a higher probability of being selected for decision by the Support Vector Machine algorithm. Pink bars represent features with a selection probability > 0.5.

**Abbreviations**: I=intercept; NEO=NEO Five Factor Inventory; S=slope; SURPS=Substance Use Risk Profile Scale; TCI=Temperament and Character Inventory.
